# *BRCA* locus-specific loss of heterozygosity in germline *BRCA1* and *BRCA2* carriers

**DOI:** 10.1038/s41467-017-00388-9

**Published:** 2017-08-22

**Authors:** Kara N. Maxwell, Bradley Wubbenhorst, Brandon M. Wenz, Daniel De Sloover, John Pluta, Lyndsey Emery, Amanda Barrett, Adam A. Kraya, Ioannis N. Anastopoulos, Shun Yu, Yuchao Jiang, Hao Chen, Nancy R. Zhang, Nicole Hackman, Kurt D’Andrea, Robert Daber, Jennifer J. D. Morrissette, Nandita Mitra, Michael Feldman, Susan M. Domchek, Katherine L. Nathanson

**Affiliations:** 1 Division of Hematology-Oncology, Department of Medicine, Perelman School of Medicine at the University of Pennsylvania, Philadelphia, PA 19104 USA; 2 Division of Translational Medicine and Human Genetics, Department of Medicine, Perelman School of Medicine at the University of Pennsylvania, Philadelphia, PA 19104 USA; 3 Department of Pathology and Laboratory Medicine, Perelman School of Medicine at the University of Pennsylvania, Philadelphia, PA 19104 USA; 4 Department of Medicine, Perelman School of Medicine at the University of Pennsylvania, Philadelphia, PA 19104 USA; 5 Department of Statistics, The Wharton School of University of Pennsylvania, Philadelphia, PA 19104 USA; 6 Department of Statistics, University of California-Davis, Davis, CA 95616 USA; 7 Department of Biostatistics, Epidemiology and Informatics, Perelman School of Medicine at the University of Pennsylvania, Philadelphia, PA 19104 USA; 8 Basser Center for BRCA and Abramson Cancer Center, Perelman School of Medicine at the University of Pennsylvania, Philadelphia, PA 19104 USA

## Abstract

Complete loss of BRCA1 or BRCA2 function is associated with sensitivity to DNA damaging agents. However, not all *BRCA1* and *BRCA2* germline mutation-associated tumors respond. Herein we report analyses of 160 *BRCA1* and *BRCA2* germline mutation-associated breast and ovarian tumors. Retention of the normal *BRCA1* or *BRCA2* allele (absence of locus-specific loss of heterozygosity (LOH)) is observed in 7% of *BRCA1* ovarian, 16% of *BRCA2* ovarian, 10% of *BRCA1* breast, and 46% of *BRCA2* breast tumors. These tumors have equivalent homologous recombination deficiency scores to sporadic tumors, significantly lower than scores in tumors with locus-specific LOH (ovarian, *P* = 0.0004; breast *P* < 0.0001, two-tailed Student’s *t*-test). Absence of locus-specific LOH is associated with decreased overall survival in ovarian cancer patients treated with platinum chemotherapy (*P* = 0.01, log-rank test). Locus-specific LOH may be a clinically useful biomarker to predict primary resistance to DNA damaging agents in patients with germline *BRCA1* and *BRCA2* mutations.

## Abstract (web summary)

Most tumours associated with germline BRCA1/BRCA2 loss of function mutations respond to DNA damaging agents, however, some do not. Herein, the authors identify that a subset of breast/ovarian tumors retain a normal allele, which is associated with decreased overall survival after DNA damage-inducing platinum chemotherapy.

## Introduction

Approximately 5% of breast and 20% of ovarian cancers arise in women carrying heterozygous germline mutations in the cancer susceptibility genes *BRCA1* or *BRCA2* ^1^. Germline *BRCA1* and *BRCA2* mutations in women are associated with an increased lifetime risk of breast and ovarian cancers^2, 3^. BRCA1 and BRCA2 are critical in double strand break repair utilizing homologous recombination (HR)^4^, disruption of which leads to high levels of genomic instability in *BRCA1* and *BRCA2* germline mutation-associated ovary^5^ and breast^6^ tumors. These tumors require additional somatic mutations, as in *TP53*, to suppress induction of DNA damage cell-cycle checkpoints, as otherwise genomic instability leads to cell-cycle arrest or apoptosis^7^.

*BRCA1* and *BRCA2* are canonical tumor suppressor genes; loss of the non-mutated (wild-type) allele at the *BRCA1* or *BRCA2* locus, termed locus-specific loss of heterozygosity (LOH) is observed in tumors^8, 9^. Cells with complete loss of BRCA1 or BRCA2 function and resultant HR-based DNA repair deficiency (HRD) have exquisite sensitivity to DNA damaging agents, such as platinum-based chemotherapeutics^10^ and PARP inhibitors^11, 12^. Tumors in *BRCA1* and *BRCA2* mutation carriers show high sensitivity to these agents in clinical trials^13–17^. The sensitivity of *BRCA*-deficient cells to platinum agents is due to the inability of cells to repair damage-induced lesions such as interstrand crosslinks^10^. PARP inhibitor sensitivity relies, in part, on a synthetic lethal interaction resulting from the inability of *BRCA*-deficient cells to repair stalled replication forks generated by PARP trapping on DNA^18, 19^. Cells with heterozygous *BRCA* mutations are significantly less sensitive to platinum agents and PARP inhibitors than cells with homozygous mutations, both in vitro^12, 20–24^ and in mouse models^24^, suggesting that complete loss of BRCA1 or BRCA2 function is a requirement for efficacy of these therapeutics. When treated with platinum chemotherapy and PARP inhibitors, individuals with and without *BRCA1* and *BRCA2* germline mutations have the same rates of adverse effects related to cell death in rapidly proliferating tissues, such as the gastrointestinal tract and hematopoietic system, further demonstrating the lack of sensitivity of heterozygous cells^15, 16^. These data support a requirement for homozygous loss of BRCA1 or BRCA2 function for sensitivity to DNA damaging agents.

Although clinical trials report excellent response rates of tumors in patients with germline *BRCA1* and *BRCA2* mutations to platinum chemotherapy and PARP inhibitors^13–17^, primary resistance has been noted^25–27^. Genomic studies have suggested that a subset of germline *BRCA1* and *BRCA2* mutation-associated tumors may not have *BRCA* locus-specific LOH^28–30^. Reversion to the heterozygous state and presumed restoration of BRCA1 or BRCA2 function has been noted as a mechanism of secondary resistance^20, 21, 31^. However, the rates of primary resistance due to maintenance of the heterozygous state (absence of locus-specific LOH) and its relationship to genomic measures and clinical outcomes are currently unknown.

We have performed an in-depth examination of the genomic profiles of primary breast and ovarian tumors in patients with germline *BRCA1* and *BRCA2* mutations with the goal of identifying correlates of therapeutic response, using two data sets. The first data set was derived from The Cancer Genome Atlas (TGCA). The second independent data set was uniformly generated from patients seen at our institution, and a tissue microarray was available for correlative studies on a subset of tumors. We show that a proportion of *BRCA1* and *BRCA2* germline mutation-associated tumors do not have locus-specific LOH. Absence of locus-specific LOH is associated with a lack of genomic measures of *BRCA*ness, and, in ovarian cancer, poorer overall survival when treated with platinum chemotherapy. We propose that locus-specific LOH may be an important clinical tool to predict primary resistance to DNA damaging agents in patients with *BRCA1* and *BRCA2* germline mutated-associated tumors.

## Results

### Establishment of analysis pipeline

We established an analysis pipeline for identification of genomic markers for BRCA1 and BRCA2 functional deficiency (termed *BRCA*ness^19^) and *BRCA* locus-specific LOH from whole exome sequencing (WES) data. Analysis of primary data from TCGA identified 100 breast and ovarian tumors with germline *BRCA1* (*n* = 55) and *BRCA2* (*n* = 45) mutations (Supplementary Fig. 1, Supplementary Data 1, Supplementary Table 1). We created a non*BRCA* tumor set (*n* = 764 breast, *n* = 215 ovary) from the remaining TCGA tumors excluding tumors with somatic mutations in, homozygous copy loss of, or transcriptional repression of *BRCA1* and/or *BRCA2*, and any breast tumors from patients who had received neoadjuvant chemotherapy.

We identified *BRCA* mutational signatures^32^ using deConstructSigs^33^ and the Somatic Signatures nonnegative matrix factorization (NMF) function^34^. When TCGA tumors were stratified by mutation and tumor type, both *BRCA1* and *BRCA2* germline mutation-associated breast and ovarian tumors had a significantly higher proportion of *BRCA* signature (Signature 3) compared to non*BRCA* breast and ovarian tumors (Supplementary Fig. 2a, b). In both analyses, there were no significant differences between any groups of *BRCA1* and *BRCA2* germline mutation-associated tumors, although non*BRCA* ovarian tumors had a significantly higher proportion of *BRCA* mutational signature than non*BRCA* breast tumors (Supplementary Fig. 2a, b). Both *BRCA1* and *BRCA2* germline mutation-associated breast and ovarian tumors had a significantly lower proportion of the aging signature (Signatures 1 and 5) compared to non*BRCA* breast and ovarian tumors (Supplementary Fig. 2c). Only a small proportion of *BRCA1* and *BRCA2* germline mutation-associated breast and ovarian tumors (*n* = 3, 3%) and non*BRCA* ovarian tumors (*n* = 8, 4%) had over 20% of mutations attributed to any signatures other than Signatures 1,3, and 5, with Signatures 2 (APOBEC) and 6 (defective mismatch repair) observed. In contrast, 110 (16%) of non*BRCA* breast tumors had over 20% of their mutations attributed to other signatures (Signatures 2, 6, 10, 13, 15, 18, or 20) (Supplementary Fig. 2d).

We developed a method to calculate genomic loss of heterozygosity (HRD-LOH)^35^, non-telomeric allelic imbalance (HRD-NtAI)^36^, and large state transitions (HRD-LST)^37^ scores from WES data using Sequenza^38^ derived allele-specific copy number (ASCN) data (Supplementary Fig. 3a–c). When tumors were stratified by mutation and tumor type, the means of these three scores (HRD-Mean) were significantly higher in *BRCA1* and *BRCA2* germline mutation-associated breast vs. non*BRCA* breast tumors, and between *BRCA1* germline mutation-associated and non*BRCA* ovarian tumors (Supplementary Fig. 3d). HRD-Mean scores were not significantly different between *BRCA1* and *BRCA2* germline mutation-associated breast and ovarian tumors. However, non*BRCA* ovarian tumors had significantly higher HRD-Mean scores than non*BRCA* breast tumors (Supplementary Fig. 3d). Of the TCGA breast and ovarian tumors, 26 and 95% of samples, respectively, underwent whole genome amplification (WGA) of tumor and/or normal DNA prior to WES. WGA is known to affect mutational profiles, copy number calls, and LOH calls^39, 40^. The proportion of mutations due to the *BRCA* signature were significantly lower and HRD-Mean scores were significantly higher for breast tumors whose DNA was prepared using WGA (*n* = 180) compared to those for which WES was performed directly from nascent DNA (*n* = 635) (Supplementary Fig. 4). Only 12 ovarian cancer TCGA samples did not have WGA performed prior to being profiled.

### *BRCA* locus-specific LOH in TCGA *BRCA1* and *BRCA2* tumors

**Fig. 1:**
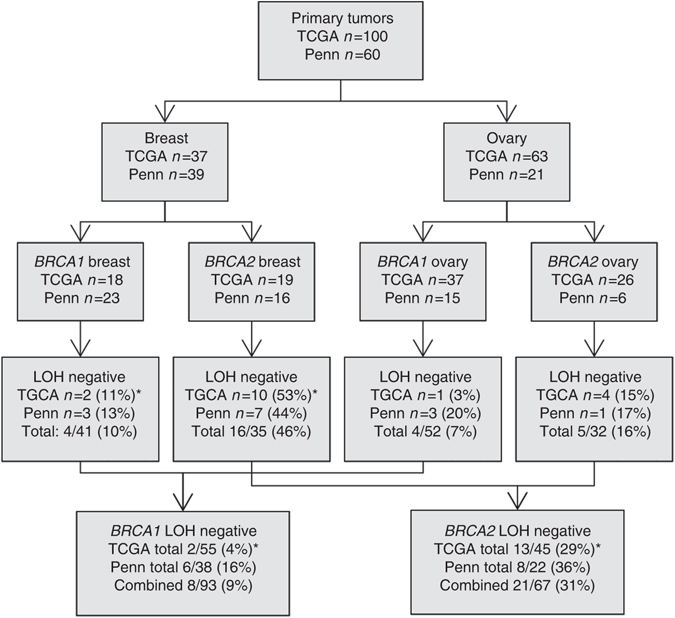
Summary of *BRCA* locus-specific LOH status of breast and ovarian tumors from individuals with germline *BRCA1* and *BRCA2* mutations. 100 tumors with germline *BRCA1* and *BRCA2* mutations were identified in The Cancer Genome Atlas (TCGA) data set and 60 tumors underwent whole exome sequencing (WES) at the University of Pennsylvania (Penn). *BRCA* locus specific loss of heterozygosity (LOH) was determined as described. *One *BRCA1* breast tumor in the TCGA had a somatic *BRCA1* mutation at 21% allele frequency and one *BRCA2* breast tumor in the TCGA had a somatic *BRCA2* mutation at 35% allele frequency. These two tumors were removed from the totals for LOH negative tumors

We next determined *BRCA* locus-specific LOH in the TCGA data set using VarScan2^41^ statistical analysis, allele frequency comparisons^28^, and Sequenza^38^ ASCN calls of the genomic region containing the *BRCA1* or *BRCA2* locus (Supplementary Data 2). Fifty-two of 55 *BRCA1* germline mutation-associated breast and ovarian tumors had locus-specific LOH (Fig. 1, Supplementary Table 2), with two breast (11%) and one ovarian (3%) tumors lacking locus-specific LOH. In contrast, 10 of 19 (53%) breast and four of 26 (15%) ovarian *BRCA2* germline mutation-associated tumors did not have locus-specific LOH (Fig. 1). The most common mechanism of locus-specific LOH was copy neutral LOH in both breast and ovarian tumors (Table 1). Tumors without *BRCA* locus-specific LOH may inactivate the wild-type allele via alternative mechanisms. One *BRCA1* and one *BRCA2* breast tumor each had somatic pathogenic mutations in the corresponding gene, at 21 and 35% allele frequency, respectively (Table 1). These two tumors were included in the group with locus-specific LOH for genomic analyses. Promoter methylation analysis of TCGA data showed that *BRCA1* promoter methylation status in ovarian and breast tumors correlates with *BRCA1* but not *BRCA2* expression levels (Supplementary Fig. 5). *BRCA1* promoter methylation as a mechanism of inactivation of the wildtype allele in the *BRCA1* tumors without locus-specific LOH was not identified.

**Table 1:** Mechanisms of biallelic loss at the germline locus in *BRCA1* and *BRCA2* mutation germline mutation-associated tumors

ASCN analysis of locus-specific LOH^a^	*BRCA1* germline mutation				*BRCA2* germline mutation			
	TCGA (*n* = 55)		Local (*n* = 38)		TCGA (*n* = 45)		Local (*n* = 22)	
LOH with deletion	9	16%	8	21%	8	18%	6	27%
Copy neutral LOH	23	42%	13	34%	15	34%	5	23%
LOH in gain	20	36%	11	29%	7	16%	4	18%
Absent locus-specific LOH	2	4%	6	16%	13	29%	8	36%
Absent LOH + somatic mutation^b^	1	2%	0	0%	1	2%	0	0%

^a^Allele-specific copy number analysis (*ASCN*) at *BRCA1* or *BRCA2* genomic locus. Categories of allele-specific copy number loss are defined as per the output of the Sequenza program: loss of heterozygosity (LOH) with deletion refers to copy number state of one with one mutant allele; copy neutral LOH refers to copy number state of two with two mutant alleles; LOH in gain refers to copy number state of ≥3 with all mutant alleles; absent locus-specific LOH refers to copy number state of ≥2 and at least one wildtype allele

^b^Identification of a somatic mutation in the corresponding gene in the tumor

### *BRCA* locus-specific LOH in Penn *BRCA1* and *BRCA2* tumors

We performed WES on nascent DNA from 60 primary untreated breast (*n* = 39) and ovarian (*n* = 21) tumors from individuals with germline *BRCA1* and *BRCA2* mutations seen at Penn Medicine. We wanted to further explore *BRCA* locus-specific LOH and its potential relationship with genomic measures of *BRCA*ness and clinical outcome in a uniformly sequenced data set, not subjected to WGA, with well-annotated clinical characteristics. Three of 23 (13%) breast and three of 15 (20%) ovarian tumors associated with *BRCA1* germline mutations, and seven of 16 (44%) breast and one of six (17%) ovarian tumors associated with *BRCA2* germline mutations did not have *BRCA* locus-specific LOH (Fig. 1, Supplementary Data 3, Supplementary Table 2). With the exception of *BRCA1* germline mutation-associated ovarian tumors, the proportion of tumors without locus-specific LOH was nearly identical in the Penn and TCGA data sets (Fig. 1). The most common mechanism of locus-specific LOH in this data set was copy neutral LOH in *BRCA1* tumors and LOH due to loss of the wild-type allele in *BRCA2* tumors (Table 1). No tumor had evidence of a second somatic mutation. Methylation specific PCR was used to analyze the *BRCA1* promoter in *BRCA1* tumors. Eight of 23 *BRCA1* germline mutation-associated breast (one without and seven with locus-specific LOH) and three of 15 *BRCA1* germline mutation-associated ovarian tumors (two without and one with locus-specific LOH) had somatic promoter methylation (Supplementary Fig. 6). Of note, the allelic distribution of promoter methylation (wildtype vs. mutant allele vs. both) is not possible using this technique.

**Fig. 2:**
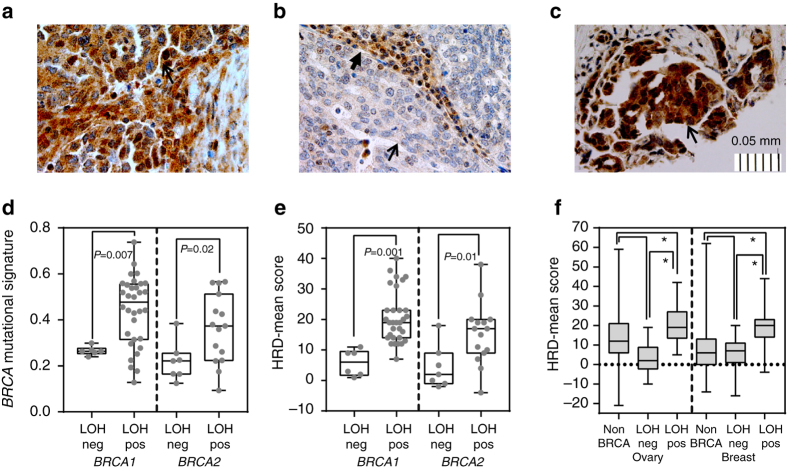
Characteristics of germline *BRCA1* and *BRCA2* breast and ovarian tumors with and without locus-specific LOH. **a** Representative immunohistochemistry for BRCA1 protein from a tumor with a *BRCA2* mutation and wildtype *BRCA1* gene. **b** Representative immunohistochemistry for BRCA1 protein from a *BRCA1* germline mutation-associated tumor with locus specific loss of heterozygosity (LOH). **c** Representative immunohistochemistry for BRCA1 protein from a *BRCA1* germline mutation-associated tumor without locus specific LOH. **d** Proportion of *BRCA* mutational signature in *BRCA1* and *BRCA2* germline mutation-associated tumors with (LOHpos) and without (LOHneg) locus-specific LOH. Data from the Penn data set is shown; data from TCGA data set can be found in the Supplementary Material. **e** Homologous recombination deficiency (HRD)-Mean scores in *BRCA1* and *BRCA2* germline mutation-associated tumors with (LOHpos) and without (LOHneg) locus-specific LOH. Data from the Penn data set is shown; data from TCGA data set can be found in Supplementary Material. **f** HRD-Mean scores in *BRCA1* and *BRCA2* germline mutation-associated ovarian and breast tumors with (LOHpos) and without (LOHneg) locus-specific LOH in combined TCGA and Penn data set. Immunohistochemistry images are shown at 40×, *open arrowheads* show clusters of tumor cells, *closed arrowheads* show infiltrating stromal tissue. The *scale bar* represents 0.05 mm at 0.01 mm increments. For all boxplots, the *center line* represents the median, *box limits* are at the 25th and 75th percentile, and *whisker limits* are at the min and max of the measured value for the represented group. Two group continuous variable comparisons were performed using a two-tailed Student’s *t*-test, *P*-values are placed on the graphs. Three group continuous variable comparisons were performed using an ordinary one-way ANOVA with Tukey’s multiple comparisons test with a single-pooled variance; *P < 0.0005

BRCA1 immunohistochemistry (IHC) was performed for 30 of 38 tumors associated with germline *BRCA1* and 13 of 22 tumors associated with germline *BRCA2* mutations. Combining tumors with both diffusely positive and heterogeneously positive nuclear staining, 12 of 13 tumors with *BRCA2* mutations had positive BRCA1 IHC; these tumors had similar BRCA1 staining in nuclei of normal stromal tissue (Table 2, Fig. 2a). In contrast, all *BRCA1*-mutated tumors with locus-specific LOH had decreased or absent BRCA1 nuclear staining compared to normal stromal cell nuclei within the tumor (Table 2, Fig. 2b, Supplementary Figs. 7 and 8). In six tumors with *BRCA1* locus-specific LOH, at least 25% of tumor nuclei retained positive BRCA1 staining, indicating intratumoral heterogeneity; two had missense mutations and four had truncating mutations in *BRCA1*. Four of the five tumors without locus-specific LOH, all with truncating mutations and including both with *BRCA1* promoter methylation, showed either diffusely or heterogeneously positive BRCA1 staining in tumor nuclei similar to normal stromal cells (Table 2, Fig. 2c, Supplementary Figs. 7 and 8). Therefore, the two tumors without *BRCA1* locus-specific LOH, but with promoter methylation, were included in the group without locus-specific LOH for genomic analyses.

**Table 2:** BRCA1 nuclear staining patterns in germline *BRCA1* and *BRCA2* mutant tumors

Germline status^a^	Total *n*	TUMOR^b^						NORMAL^b^					
		Positive		Het Positive		Negative		Positive		Het Positive		Negative	
		*n*	*%*	*n*	*%*	*n*	*%*	*n*	*%*	*n*	*%*	*n*	*%*
*BRCA1*, LOHpos	25	0	0	6	24	19	76	10	40	5	20	10	40
*BRCA1*, LOHneg	5	1	20	3	60	1	20	3	60	1	20	1	20
*BRCA2*	13	10	77	2	16	1	7	5	38	5	38	3	23

^a^LOHpos refers to presence of locus-specific LOH LOHneg refers to absence of locus-specific LOH

^b^For all samples, immunohistochemical staining for BRCA1 was graded from 0 to 3+ in three cores in both tumor nuclei and normal tissue nuclei with mutation status blinded. The maximum score is shown for nuclear staining. Positive nuclear staining was defined as 100% of nuclei with at least 1+ staining in all three cores. Heterogeneous positive staining was defined as >25% of nuclei with at least 1+ staining in at least one of three cores

### *BRCA* locus-specific LOH and genomic signatures of *BRCA*ness

Tumors in the Penn data set, all sequenced from nascent DNA, had a similar distribution of mutational signatures (majority Signatures 1 and 3) as the TCGA *BRCA1/2* germline mutation-associated tumors. Tumors in the Penn data set without locus-specific LOH had a significantly lower proportion of *BRCA* mutational signature (*BRCA1*, *P* = 0.007 and *BRCA2*, *P* = 0.02) compared to tumors with locus-specific LOH, when mutational signatures were analyzed by both NMF and deconstructSigs (Fig. 2d). No significant differences in proportion of mutational signature were observed in the TCGA data set; WGA may alter these profiles (Supplementary Fig. 9a). Tumors without locus-specific LOH in both data sets trended towards having higher proportions of mutations due to Signature 1 (aging) (Supplementary Fig. 9b, c). Five tumors without locus-specific LOH had evidence of either Signature 2 or 13 (APOBEC)-associated mutations (Supplementary Fig. 9d, e, Supplementary Table 2).

Tumors without locus-specific LOH in the Penn data set had significantly lower HRD-Mean scores compared to tumors with locus-specific LOH for *BRCA1* (*P* = 0.001) and *BRCA2* (*P* = 0.01) (Fig. 2e). Similar results were seen in the TCGA data set for both *BRCA1* (*P* < 0.0001) and *BRCA2* (*P* = 0.01) (Supplementary Fig. 10a). When *BRCA1* and *BRCA2* tumors in both data sets were combined, despite differences in sequencing platform, both ovarian and breast tumors associated with germline *BRCA1* and *BRCA2* mutations without locus-specific LOH had significantly lower HRD-Mean scores compared to ovarian and breast tumors with locus-specific LOH (*P* < 0.0005) (Fig. 2f). HRD-Mean scores in tumors without locus-specific LOH were similar to non*BRCA* tumors (Fig. 2f). To exclude a possible effect of WGA samples on this result, the combined Penn and TCGA breast data were reanalyzed including only TCGA tumors derived from nascent DNA (all Penn tumors were derived from nascent DNA). In this analysis, HRD-Mean scores remained significantly higher in tumors with locus-specific LOH compared to both tumors without locus-specific LOH and non*BRCA* tumors (*P* < 0.0001, Supplementary Fig. 10b).

### Molecular correlates of *BRCA* locus-specific LOH

We evaluated whether somatic mutations differed in *BRCA1* and *BRCA2* germline mutation-associated tumors with and without locus-specific LOH. We identified single nucleotide and insertion/deletion somatic mutations in the 60 Penn and 100 TCGA tumors. Mutational burden was defined as the number of somatic nonsynonymous mutations per megabase^42^. In the Penn data set, *BRCA1* germline mutation-associated tumors had an average of 0.96 ± 0.39 mutations/Mb and *BRCA2* germline mutation-associated tumors an average of 1.08 ± 1.00 mutations/Mb, consistent with mutational burden in studies of TCGA *BRCA1* and *BRCA2* germline mutation-associated ovarian tumors^43^. *BRCA1* germline mutation-associated tumors, with and without locus-specific LOH, had similar mutational burdens (0.78 ± 0.20 vs. 1.00 ± 0.07, comparison not significant). However, *BRCA2* germline mutation-associated tumors without locus-specific LOH had a significantly lower mutational burden compared to those with LOH (0.37 ± 0.11 vs. 1.41 ± 0.27, *P* = 0.02). Mutational burden was similar between tumors with and without locus-specific LOH in the TCGA data set (0.92 ± 0.36 vs. 1.29 ± 0.06, comparison not significant for *BRCA1* and 0.99 ± 0.13 vs. 1.34 ± 0.11, *P* = 0.09 for *BRCA2*).

**Fig. 3:**
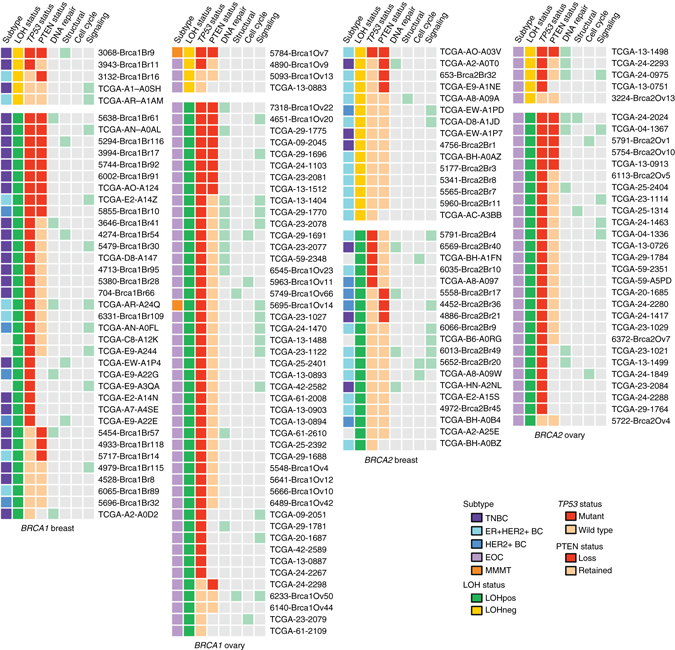
Recurrent somatic mutations in *BRCA1* and *BRCA2* germline mutation-associated breast and ovarian tumors. Profile of major classes of cancer genes (defined as COSMIC cancer gene census genes and/or as reported^44^) with somatic mutations identified in *BRCA1* and *BRCA2* germline mutation-associated breast and ovarian tumors with and without locus specific LOH (LOHpos and LOHneg, respectively) in the Penn and TCGA data sets. Likely pathogenic/pathogenic mutations in genes classified by DAVID as genes involved in DNA repair, cell structure genes, cell cycle regulation genes, and oncogenic signaling genes are marked by *light green boxes*. *LOH* loss of heterozygosity, *BC* breast cancer, *TNBC* triple negative breast cancer, *ER* estrogen receptor, *EOC* epithelial ovarian cancer, *MMMT* carcinosarcoma. *TP53* status refers to mutation presence (mutant) or absence (wildtype). *PTEN* loss is as described in the Results

MutSigCV^42^ analysis identified only *TP53* (*q* = 0) and *PTEN* (q = 0.006) as significantly mutated genes in Penn *BRCA1* and *BRCA2* germline mutation-associated tumors (Supplementary Data 4). In the TCGA *BRCA1* and *BRCA2* germline mutation-associated tumors, *TP53* (*q* = 3.1*e* ^−12^) and *RB1* (q = 2.2*e* ^−5^) were significantly mutated; *PTEN* had *q* = 0.2 (Supplementary Data 5). Both *BRCA1* and *BRCA2* germline mutation-associated tumors without locus-specific LOH were significantly less likely to have a *TP53* mutation than *BRCA1* and *BRCA2* tumors with locus-specific LOH (44 vs. 84%, *P* = 0.01 for *BRCA1*; 24% vs. 68%, *P* = 0.001 for *BRCA2*). Beyond *TP53* and *PTEN*, 41% of *BRCA1* and *BRCA2* germline mutation-associated tumors had a likely pathogenic/pathogenic mutation in a cancer gene (defined as COSMIC cancer gene census gene and/or as reported^44^) (Fig. 3). Across the 160 tumors, six cancer genes had mutations in more than two tumors: *NF1* (8), *PIK3CA* (6), *RB1* (6), *ARID1A* (4), *TDG* (4), and *ERCC6* (3). There were no differences in the spectrum of cancer gene mutations between tumors with and without locus-specific LOH (Fig. 3, Supplementary Table 3).

*PTEN* loss is a common feature of breast and ovarian tumors associated with germline mutations in *BRCA1* and *BRCA2* ^45^. *PTEN* allele-specific copy number status by WES was compared to PTEN status by IHC on 44 Penn tumors so that PTEN status in tumors without IHC could be estimated (Supplementary Fig. 11, Supplementary Table 4). Using this method, the rate of *PTEN* loss did not differ significantly between tumors with and without locus-specific LOH for *BRCA1* and *BRCA2* (38 vs. 30%, and 32 vs. 21%, respectively).

### Clinical correlates of *BRCA* locus-specific LOH

**Fig. 4:**
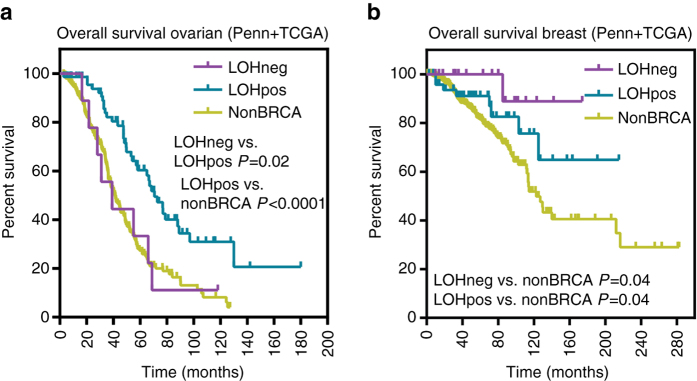
Overall survival based on locus specific LOH status in patients with germline *BRCA1* and *BRCA2* mutations. **a** Kaplan–Meier overall survival curve for patients with *BRCA1* and *BRCA2* germline mutation-associated ovarian tumors with (LOHpos) and without (LOHneg) locus specific loss of heterozygosity (LOH) in the TCGA and Penn data set. Survival proportions compared using a log-rank (Mantel–Cox) test. LOHneg vs. non*BRCA* comparisons were nonsignificant. **b** Kaplan–Meier overall survival curve for patients with *BRCA1* and *BRCA2* germline mutation-associated breast tumors with (LOHpos) and without (LOHneg) locus specific LOH of the in the TCGA and Penn data set. Survival proportions compared using a log-rank (Mantel–Cox) test. LOHneg vs. LOHpos comparisons were nonsignificant

We next evaluated the association of *BRCA* locus-specific LOH with clinical characteristics. No significant association between *BRCA* locus-specific LOH status and age of cancer diagnosis, breast tumor size, node positivity, or hormone receptor status or with ovarian tumor grade was observed (Supplementary Table 5). In ovarian cancer patients treated with adjuvant platinum based chemotherapy, patients whose tumors lacked *BRCA* locus-specific LOH had a significantly worse overall survival compared to patients whose tumors had locus-specific LOH, similar to non*BRCA* patients (Fig. 4a). Absence of locus-specific LOH remained significantly associated with overall survival using a Cox proportional hazard model to control for site (Penn vs. TCGA), stage at diagnosis, and gene (*BRCA1* vs. *BRCA2*) (Supplementary Fig. 12a, b). In breast cancer patients, absence of locus-specific LOH was associated with better survival compared to non*BRCA* patients but was similar to patients whose tumors had locus-specific LOH (Fig. 4b). However, this was likely due to an enrichment of Stage III and/or triple negative patients in the non*BRCA* group (Supplementary Fig. 12c).

## Discussion

It has been previously assumed that all tumors associated with germline *BRCA1* and *BRCA2* mutations have locus-specific LOH, and therefore loss of BRCA1 or BRCA2 function. However, large-scale studies have suggested that not all tumors from individuals with germline *BRCA1* or *BRCA2* mutations have locus-specific LOH^28–30^, and we hypothesized the absence of locus-specific LOH may be a biomarker of primary resistance to platinums and PARP inhibitors.

In the TGCA and Penn data sets, most tumors with germline mutations associated with *BRCA1* had locus-specific LOH. Only nine of 93 (10%) of *BRCA1* tumors in the combined data set lacked locus-specific LOH. All of the *BRCA1* germline mutation-associated tumors without locus-specific LOH had HRD-Mean and *BRCA* mutational signature scores well below the mean for tumors demonstrating locus-specific LOH. Thus, a small percentage of *BRCA1* germline mutation-associated tumors do not display locus-specific LOH and lack a *BRCA*ness phenotype. Of the nine tumors lacking *BRCA1* locus-specific LOH, three came from patients with a history of receiving chemotherapy for a prior cancer, and three were tumors of types not typically associated with *BRCA1* germline mutations (an ovarian carcinosarcoma, one ER+ breast ductal carcinoma, and one ER+ breast lobular carcinoma), suggesting that second primary and non-classic histological tumors may be enriched for absence of locus-specific LOH.

We evaluated whether the presence or absence of locus-specific LOH of *BRCA1* was associated with retention of the protein as measured by BRCA1 IHC. All tumors demonstrating locus-specific LOH had decreased BRCA1 protein as compared to the surrounding stroma, with most tumors demonstrating complete absence of BRCA1 protein. However, of note, a subset of tumors with *BRCA1* locus-specific LOH retained BRCA1 expression in at least 25% of nuclei. In contrast, in 80% (four of five) of tumors without *BRCA1* locus-specific LOH, BRCA1 protein was retained; of these two also had *BRCA1* promoter methylation. Although promoter methylation of the wildtype allele would be expected to result in absence of protein, these data are consistent with prior studies showing approximately 20% of tumors with *BRCA1* promoter methylation have retained BRCA1 protein^46–48^, which could be due to promoter methylation of the mutant as opposed to the wildtype allele. Overall, these results suggest that *BRCA1* locus-specific LOH as measured genetically is a marker for protein expression. However, we found that tumors associated with *BRCA1* mutations demonstrate heterogeneity in protein expression, and thus bulk sequencing, promoter methylation and LOH analysis may mask sub-populations of tumor cells that vary in regards to *BRCA1* locus-specific LOH.

Almost identical percentages of tumors associated with *BRCA2* germline mutations in the TCGA and Penn set did not have locus-specific LOH. Absence of locus-specific LOH was found at a particularly high rate for *BRCA2* germline mutation-associated breast tumors (46%). Over 75% of *BRCA2* germline mutation-associated tumors without locus-specific LOH had HRD scores significantly below the mean for tumors demonstrating locus-specific LOH. Additionally, we identified molecular correlates associated with absence of locus-specific LOH, also consistent with a lack of *BRCA*ness, including a lower mutational burden in tumors associated with *BRCA2* germline mutations and a significantly decreased frequency of *TP53* mutations in tumors associated with *BRCA1* and *BRCA2* germline mutations.

Tumors without locus-specific LOH in patients with *BRCA1* or *BRCA2* germline mutations may arise due to various mechanisms of carcinogenesis. Several tumors arose in patients previously treated with chemotherapy and so subjected to other types of DNA damage which may induce tumor formation irrespective of germline *BRCA* status, although breast and ovarian cancers are not typically considered chemotherapy-induced malignancies. *BRCA1* and *BRCA2* mutant cells may be more sensitive in general to chemotherapy^49^ which could thereby eliminate precancerous clones promoting growth of tumors that are not fully deficient in BRCA function. Tumors also may develop through mechanisms similar to sporadic breast and ovarian tumors, related to estrogen exposure or aging, for example. Supportive of this postulate is a similar rate of an absence of locus-specific LOH in *BRCA2* carriers in the two independent tumor sets, similar HRD-Mean scores to non*BRCA* tumors and higher percentage of aging signature in those tumors without *BRCA* locus-specific LOH in the Penn data set. However, age of cancer diagnosis was not associated with LOH status and seven of 30 (23%) of the tumors without *BRCA* locus-specific LOH developed in patients under age 40, and 50% under age 50. As breast and ovarian cancers diagnosed under age 50 are rare in the general population, these data suggest possible alternate mechanisms of tumoriogenesis related to the underlying inherited mutation. *BRCA1* and *BRCA2* heterozygous mutant states have been shown to contribute to tumor formation in pancreatic and ovarian cancer mouse models^50, 51^, consistent with studies that demonstrate that haploinsufficiency for BRCA1 and BRCA2 leads to multiple levels of cellular dysfunction^29, 52–54^. Radiation-induced tumors in heterozygous *Brca1* mice do not demonstrate locus-specific LOH^55^. It is therefore possible that tumors without locus-specific LOH in *BRCA1* and *BRCA2* germline mutation carriers develop due to haploinsufficiency of BRCA1 or BRCA2 function^56^ in conjunction with other predisposing events, such as environmental exposures.

*BRCA1* and *BRCA2* germline mutation status is known to be associated with improved overall survival in ovarian cancer patients^57^. We found that both *BRCA1* and *BRCA2* ovarian tumors without locus-specific LOH treated with adjuvant platinum based chemotherapy have lower overall survival than tumors with locus-specific LOH, at rates similar to sporadic tumors. This finding is consistent with preclinical data demonstrating that both human cell lines and mouse models lacking BRCA1 or BRCA2, due to biallelic mutations or knock out, are responsive to platinum and PARP inhibitors, whereas those that retain some level of BRCA1 or BRCA2 function are not^12, 20–24^. Our data support that *BRCA* locus-specific LOH is necessary for tumors in *BRCA1* and *BRCA2* germline mutation carriers to respond to platinum therapy, and potentially by extension PARP inhibitors.

There are limitations to the current study. We are using locus-specific LOH status to extrapolate BRCA1 or BRCA2 function. As our data suggest the presence of intratumoral heterogeneity protein expression of tumors associated with germline *BRCA1* and *BRCA2* mutation carriers, it is possible that further information may be obtained by single cell analysis. Sub-populations of cells within tumors may vary in terms of *BRCA* locus-specific LOH, and thus responsiveness to DNA damaging agents.

In aggregate, our findings from the largest single study of breast and ovarian tumors associated with *BRCA1* and *BRCA2* germline mutations suggest that locus-specific LOH may be a biomarker for a *BRCA*ness phenotype in whole tumor formalin-fixed paraffin embedded (FFPE) specimens. There are other recently published *BRCA*ness scores^58, 59^ which may predict therapy response. However, these scores require additional tumor genomic testing using single nucleotide polymorphism (SNP) arrays^59^ or whole genome sequencing^58^. Locus-specific LOH, which can be determined from already existing DNA-based FFPE tumor testing pipelines remains a clinically promising and more cost-effective means of predicting therapy response in germline *BRCA1* and *BRCA2* carriers. Our findings need to be confirmed prospectively, particularly in breast cancer patients stratified for stage and therapy, but provide support for the use of a *BRCA* locus-specific LOH assay to predict primary response to platinums, and potentially PARP inhibitors, in patients with germline *BRCA1* and *BRCA2* mutations. Such an assay could have broad applicability as germline *BRCA1* and *BRCA2* mutations have been associated with 3% of metastatic tumors of multiple subtypes^60, 61^. These results also emphasize that tumors associated with germline *BRCA1* and *BRCA2* mutations, and likely tumors in other carriers of mutations in DNA damage response genes, should not be considered uniformly. Further study is needed to investigate the precise role of the individual inherited mutations in DNA damage response pathway genes in the development of cancer, and whether or not locus-specific LOH may be useful to predict response to DNA damaging agents in carriers of mutations in other DNA damage response genes.

## Methods

### Identification of TCGA tumors with germline ***BRCA1*** and ***BRCA2*** mutations

Primary (Level 1) WES data was obtained to create the TCGA dat sets. To access the Level 1 DNA sequencing data for breast and ovarian tumors from TCGA, a project request was submitted and approved by the National Center for Biotechnology Information Genotypes and Phenotypes Database (NCBI dbGaP) Data Access Request system, Protocol #5309 “BRCA1 and BRCA2 mutations in breast and ovarian cancer”. Breast and ovarian samples potentially with controlled-access Level 1 WES binary alignment (.bam) files were identified from the Genomic Data Commons (GDC) (https://gdc.cancer.gov/) (*n* = 1098 breast tumors and 590 ovarian tumors) (Supplementary Fig. 1). After filtering for samples with WES data not available on the GDC commons (*n* = 406) and samples not passing our sequencing pipeline quality control analysis (*n* = 104), 1178 tumor/normal pairs were subjected to downstream analysis. Germline *BRCA1* and *BRCA2* mutations were identified using VarScan2^41^ (http://dkoboldt.github.io/varscan/); and from the normal variant calls of Mutect^62^ (https://github.com/broadinstitute/mutect) and subjected to variant classification^63^. Tumors were determined to be associated with germline *BRCA1* and *BRCA2* mutations (*n* = 100) if they met the following criteria: (1) known pathogenic *BRCA1* or *BRCA2* mutation as per ENIGMA classification in germline and tumor sample; (2) germline allelic fraction (AF) >0.30; and (3) total depth >30 in germline and tumor at the mutation locus. Germline mutations were confirmed by review of traces in the Integrated Genome Viewer IGV (http://software.broadinstitute.org/software/igv/). We thus curated a data set of 100 *BRCA1* and *BRCA2* germline mutation-associated breast and ovarian tumors from the TCGA consisting of 37 breast (18 *BRCA1* and 19 *BRCA2*) and 63 ovarian tumors (37 *BRCA1* and 26 *BRCA2*).

### Identification of non***BRCA*** TCGA tumors

To create a non*BRCA* tumor set from the remaining 1078 tumor/normal BAM pairs (Supplementary Fig. 1), we analyzed primary WES data using Mutect^62^, VarScan2^41^, and Sequenza^38^ (https://cran.r-project.org/web/packages/sequenza/index.html). Tumors were excluded if they were found to have: (1) a pathogenic somatic *BRCA1* or *BRCA2* mutation (*n* = 23 breast and *n* = 18 ovarian) and/or (2) homozygous copy number deletion of *BRCA1* or *BRCA2* (*n* = 11 breast and *n* = 4 ovarian). Finally, Level 3 RNAseq *z*-scores, microarray *Z*-scores and HK27/HK450m methylation beta values were bulk downloaded from The Cancer Genomics Hub of the University of Santa Cruz (https://cghub.ucsc.edu/, project now completed) for the tumor/normal pairs. Samples were excluded with *z*-scores < −1.5 and HK450m>0.5 for *BRCA1* and RNAseq *z*-score < −1.5 for *BRCA2* expression (*n* = 12 breast and 20 ovarian). Breast tumors were also excluded from patients who had received neoadjuvant chemotherapy (*n* = 11 breast).

### Sample acquisition and preparation of Penn germline ***BRCA1*** and ***BRCA2*** mutated tumor set

The Penn study population was ascertained from academic and community hospital sites within Penn Medicine and the Penn Cancer Network. Acquisition of the patient samples was approved by the Institutional Review Board of the University of Pennsylvania, and informed consent was obtained from each participant for use of their samples and clinical data in genetic studies. Eligibility criteria for the study were (1) diagnosis of breast or ovarian cancer; (2) positive *BRCA1/2* sequencing in a Clinical Laboratory Improvement Amendments-approved laboratory; and (3) available archived blood DNA and FFPE tumor blocks. Two hundred and twenty-three patients with breast and ovarian tumors meeting criteria were identified and 151 blocks were available or received by a central Pathology core (Supplementary Fig. 1). FFPE tumor blocks were sectioned and stained with hematoxylin and eosin to ensure sections of over 70% invasive tumor were used for DNA extraction, 68 tumors failed pathology quality control. Selected tumor areas of slides or rolls were dissolved in Deparaffinization Solution (Qiagen) and purified using Gentra PureGene Reagents (Qiagen) following manufacturer's protocols. All DNA samples were quantitated with a Qubit and tumor DNA was subjected to a quantitative PCR based QC Kit (Kapa Biosystems) for analysis of DNA quality (sample QC), and 23 samples failed laboratory quality control. This process resulted in a set 60 tumors, 38 from *BRCA1* (23 breast, 15 ovarian tumors) and 22 from *BRCA2* (16 breast, six ovarian tumors) mutation carriers (Supplementary Fig. 1). Germline DNA from blood or saliva was extracted using standard protocols in the laboratory. Library preparation of tumor and matched germline DNA was as described^64, 65^. In all Penn cases, nascent DNA, not subjected to WGA, was used for WES. Details of the Penn population are found in Supplementary Table 1 and summarized in Supplementary Table 2. Libraries were subjected to WES using the Agilent All-Exon Kit v5 (Santa Clara, CA). Tumors were sequenced on an Illumina Hi-Seq 2000 to an average mean depth of 141× and matched blood DNA was sequenced to an average mean depth of 155×.

### Bioinformatics analysis and identification of somatic mutations in the Penn and TCGA tumor set

Quality control measures were determined with Picard Tools (https://broadinstitute.github.io/picard/). Penn tumors and matched germline were aligned to the hg19 assembly of the human genome using Burrows–Wheeler Aligner for short-read alignment^66^ (http://bio-bwa.sourceforge.net/). Variants underwent initial quality control filtering according to Genome Analysis ToolKit (GATK)^67^ best practices. Downloaded TCGA BAM files had been aligned to the hg38 assembly of the human genome. All exonic single nucleotide and insertion/deletion variants were identified using a combination of GATK^67^ (https://software.broadinstitute.org/gatk/), Mutect^62^, and VarScan2^41^. Variants identified by initial bioinformatic analysis were annotated using ANNOVAR^68^ (http://annovar.openbioinformatics.org/en/latest/). Exonic variants were kept as somatic variants if they met the following criteria: (1) alternate allele depth in germline less than five reads; (2) population frequency <1% in EVS6500 (http://evs.gs.washington.edu/EVS/) and 1000 genomes (http://www.1000genomes.org/) databases; (3) not found in genomic regions of high (>89%) segmental duplications; (4) not categorized as synonymous variants; and (5) alternate allele supported by greater than 10 reads in the tumor. Manual curation of variants using a custom derived pipeline^64^ was used to classify variants as deleterious (D), likely deleterious (LD), variants of uncertain significance (VUS), likely benign and benign (B). Somatic mutations were defined as D or LD variants and rare VUS (variants with population allele frequency <0.1%) in the tumors. MutSigCV (http://software.broadinstitute.org/cancer/software/)^42^ was used to identify significantly mutated genes. Cancer genes are those in the COSMIC cancer gene census (http://cancer.sanger.ac.uk/census/) and/or reported^44^.

### Determination of ***BRCA*** locus-specific loss of heterozygosity in Penn and TCGA data sets

A combination of VarScan2^41^, allele frequency comparisons, and allele-specific copy number calls were used to determine *BRCA* locus-specific LOH. Estimates of tumor purity (cellularity) were determined using Sequenza and inputted into VarScan2 variant calling. The sample was assigned a locus-specific LOH positive status if the VarScan2 somatic *P*-value was significant and a locus-specific LOH negative status if the VarScan2 germline *P*-value was significant. Allele-specific copy number calls of the genomic region containing the *BRCA1* or *BRCA2* mutation were determined by Sequenza. The copy number of the genomic region surrounding the germline *BRCA1* or *BRCA2* mutation (CN) and the number of mutant (*m*) alleles as per the output of the Sequenza program were used to assign two states of absent locus-specific LOH—heterozygous diploid (CN = 2;*m* = 1) or amplified with gain of non-mutant (wildtype) allele (CN>2;*m* = 1)—and three states of locus-specific LOH—loss (CN = 1;*m* = 1), copy neutral LOH (CN = 2;*m* = 2), and amplified with LOH (CN>2;*m*>2). The genomic regions surrounding the germline *BRCA1* and *BRCA2* mutation ranged from less than one to over 100 Mb in length. In cases where the VarScan2 and ASCN calls differed (six of 100 TCGA tumors and four of 60 Penn tumors), the difference between cellularity corrected tumor allele frequency and blood allele frequency (ΔAF) was determined; the sample was assigned a locus-specific LOH positive status if ΔAF>0.20^28^. Finally, five of 100 TCGA tumors had a germline *BRCA1* or *BRCA2* mutation identified only by normal variant calling by Mutect; for these a combination of ASCN and ΔAF was used to determine locus-specific LOH.

### Determination of mutational signatures in the Penn and TCGA data sets

Somatic mutations for determination of mutational signatures in the local and TCGA data set were identified using the MuTect2 variant calls derived from BAM files obtained as above. Mutational signatures were then extracted with the SomaticSignatures program^34^ using *r* = 4 and NMF options simultaneously for *n* = 80 Penn breast and ovarian tumors and *n* = 1186 TCGA breast and ovarian tumors. In addition, the deconstructSigs program was additionally used to determine the proportion of known mutational signatures in the data^33^. Using deconstructSigs, 60 TCGA tumors had greater than 30% of their mutational signatures due to sequencing artifact (signature R1–R3, U1–U2)^32^, including one *BRCA2* tumor without locus-specific LOH, three *BRCA1* tumors with locus-specific LOH, seven non*BRCA* ovarian, and 49 non*BRCA* breast tumors. These tumors were therefore excluded from all subsequent genomic analyses. No Penn tumors had greater than 10% of their mutational signatures due to sequencing artifact. Mutational signatures were calculated blinded to locus-specific LOH status.

### Determination of homologous recombination deficiency in the Penn and TCGA data sets

Allele-specific copy number states were determined using Sequenza and used to calculate the HRD scores NtAI^36^, LST^37^, and genomic LOH (HRD-LOH)^35^ using custom R-scripts, which are available upon request. Non-telomeric allelic imbalance (NtAI) scores were derived from the Sequenza data by summing the number of segments of allelic imbalance that were post-centromeric to the sub-telomeric regions and >11 Mb in length. Large state transition scores were derived from the Sequenza data by summing the number of breakpoints creating >3 Mb segments that were >10 Mb from one another. Raw LST scores were corrected for ploidy (LSTm) using the equation LSTm = LST–15.5 × ploidy. HRD-LOH scores were derived from Sequenza data by summing the number of segments of LOH >15 Mb in length excluding segments found on Chromosome 17. HRD scores were calculated blinded to locus-specific LOH status.

### Methylation specific PCR analysis of the ***BRCA1*** promoter

Methylation specific PCR analysis was performed using primers specific for unmethylated and methylated *BRCA1* promoter as described^69, 70^. Primers were obtained from Integrated DNA Technologies (Coralville, IA). Bands were resolved on a 6% Tris-buffered EDTA (TBE) gel from Invitrogen (Carlsbad, CA). A representative full blot is found in Supplementary Fig. 6. Band intensity of samples was compared to a fully methylated and an unmethylated control and a band intensity ≥ two-fold of the band seen in the unmethylated control was considered positive for *BRCA1* promoter methylation. Constitutional blood DNA was concurrently tested and demonstrated an absence of constitutional methylation of the *BRCA1* promoter in tumors with positive *BRCA1* promoter methylation. Methylation was quantified blinded to locus-specific LOH status.

### PTEN and BRCA1 immunohistochemistry

Tissue microarrays containing 44 of the sequenced tumors were made. Sections were prepared of tumor and surrounding normal tissue in triplicate for each sample available. Immunohistochemistry was performed for BRCA1 using MS110 antibody from EMD Millipore (Billerica, Massachusetts), staining for one tumor failed. Immunohistochemistry was performed for PTEN using D4.3 antibody from Cell Signaling Technology (Danvers, Massachusetts). Scoring for immunohistochemical stains was performed in triplicate for each tissue sample. Stain intensity was graded on a four-point scale (0–3) by a board-certified pathologist (A.B., M.F.), with 0 = no staining, 1 = weak or partial staining, 2 = moderate staining, 3 = strong staining. *BRCA1* and *BRCA2* mutation and LOH status and *PTEN* allele-specific copy number status was blinded. Two cell populations (tumor and non-tumor cell types) were analyzed in each sample, and the intensity of cytoplasmic and nuclear staining in each population was assessed independently. In addition, the percentage of cells with the highest intensity of staining was also reported.

### Use of allele-specific copy number calls at the genomic ***PTEN*** locus to extrapolate PTEN status

To determine if *PTEN* allele-specific copy number status by Sequenza^38^ based WES analysis accurately predicted protein loss, we analyzed PTEN status by IHC on 44 tumors from the Penn data set as above (Supplementary Fig. 11). All 10 tumors with *PTEN* copy number loss (*n* = 7) or a truncating *PTEN* mutation with copy neutral LOH (*n* = 3) did not stain positively for PTEN on IHC (Supplementary Table 4). Twenty-four of 25 tumors that had wildtype *PTEN* with a copy number state of two or more had staining of PTEN on IHC (concordance 97%). Of nine tumors without an identifiable mutation but copy neutral LOH of *PTEN* on IHC: four stained positively and five did not stain for PTEN. Given these results, for the 16 Penn tumors without available IHC data and the 100 TCGA tumors, the ASCN state of the *PTEN* genomic locus was used to extrapolate PTEN status. *PTEN* copy number loss or truncating mutation was used to extrapolate PTEN loss (*n* = 15), and *PTEN* wildtype status at copy number two or more to extrapolate retention of PTEN (*n* = 64). Eleven tumors with copy neutral LOH were excluded from analysis.

### Statistical and clinical data analyses

Means of continuous variables were compared using a two-tailed Student’s *t*-test. Outliers were excluded based on Grubb’s test (extreme studentized deviate test). Comparisons of rates in different groups were determined using a two-tailed Fisher’s exact test of significance for two groups or a one-way ANOVA with correction for multiple comparisons for three or more groups. Clinical data were obtained for the patients in the Penn data set by IRB approved chart review. The overall survival time for all patients was determined from the date of diagnosis to the time of last follow-up or death by query of the medical record for each Penn patient or by bulk data download from The Cancer Genomics Hub of the University of California at Santa Cruz (https://cghub.ucsc.edu/, project now completed) for the TCGA data set. Patients alive at the end of follow-up were censored. Survival was compared using a log-rank (Mantel–Cox) test. In addition, a Cox proportional hazards model was used to examine the effect of locus-specific LOH status, site (TCGA vs. Penn) and stage at diagnosis for ovarian cancer patients. For breast cancer patients, a Cox proportional hazards model was used to examine the effect of locus-specific LOH status, site (TCGA vs. Penn), stage at diagnosis, and hormone receptor status. Kaplan–Meier curves were constructed for each analysis.

### Data availability

The WES data that supports this study have been deposited in the National Center for Biotechnology Information (NCBI)’s Sequence Read Archive (SRA, https://www.ncbi.nlm.nih.gov/sra) with BioProject ID PRJNA38804 and can be accessed at: http://www.ncbi.nlm.nih.gov/bioproject/388048. The TCGA data is available from the National Cancer Institute’s Genome Data Commons (https://gdc.cancer.gov/). The remaining data are available within the article and its Supplementary Information files or available from the authors upon request.

## Electronic supplementary material

Supplementary Information

Supplementary Data 1

Supplementary Data 2

Supplementary Data 3

Supplementary Data 4

Supplementary Data 5
