# Supplementary material for: BRCA locus-specific loss of heterozygosity in germline BRCA1 and BRCA2 carriers
Source: Nat Commun. 2017 Aug 22;8:319. doi: 10.1038/s41467-017-00388-9 (PMC5567274; doi:10.1038/s41467-017-00388-9)
Supplement: Supplementary file 1 — Supplementary Information [file 41467_2017_388_MOESM1_ESM.pdf]

File Name: Supplementary Information
Description: Supplementary Figures and Supplementary Tables.

File Name: Supplementary Data 1
Description: Clinical data for BRCA1 and BRCA2 germline mutation-associated tumors analyzed by whole exome sequencing

File Name: Supplementary Data 2
Description: Locus-specific LOH data for TCGA BRCA tumors

File Name: Supplementary Data 3
Description: Locus-specific LOH data for Penn BRCA tumors

File Name: Supplementary Data 4
Description: MutSigCV Analysis of the Penn BRCA dataset

File Name: Supplementary Data 5
Description: MutSigCV Analysis of TCGA BRCA dataset

a. TCGA Dataset

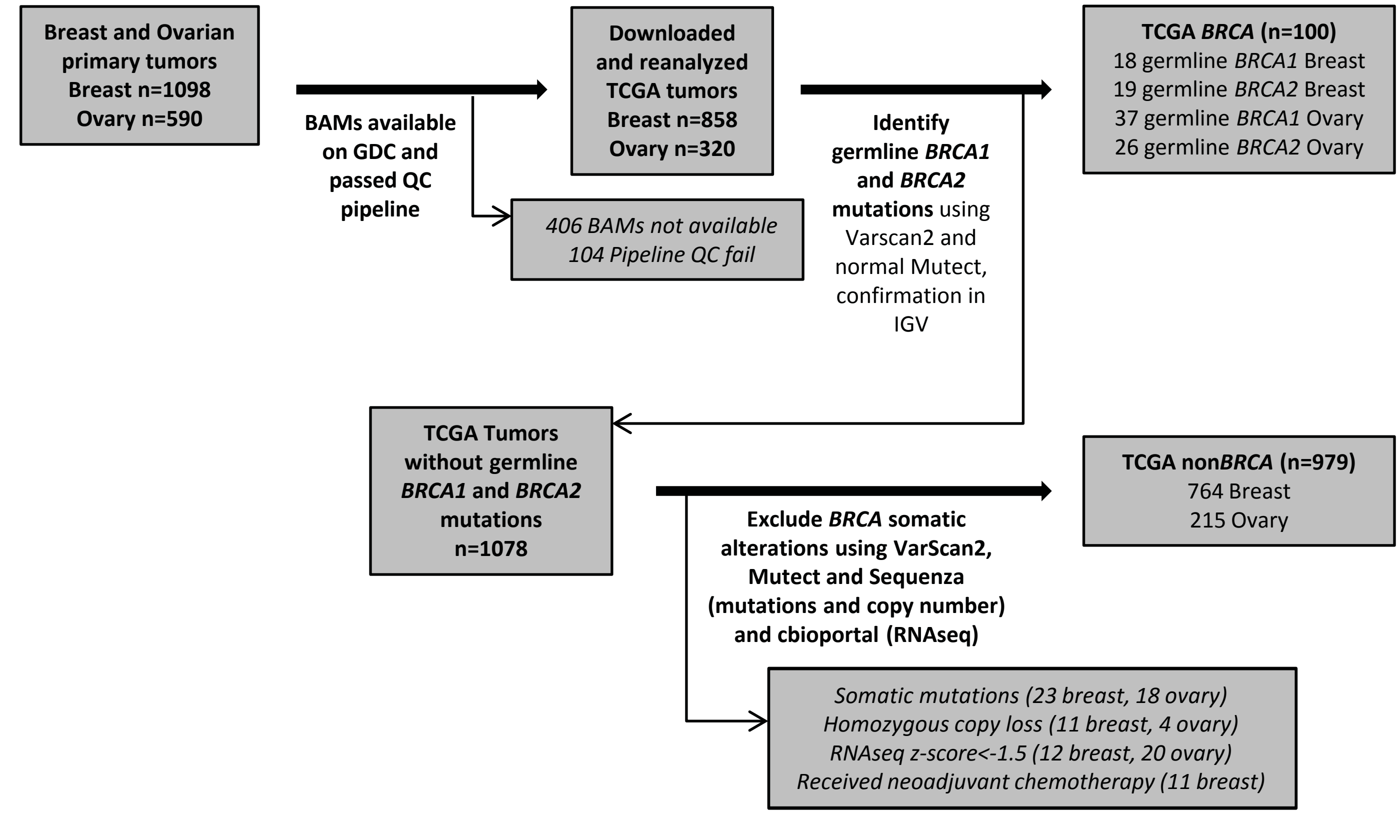

b. Penn Dataset

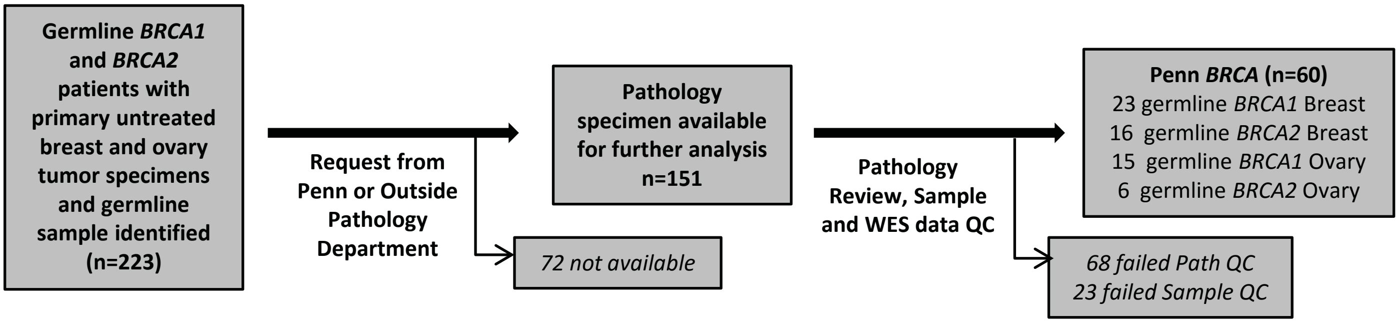

Supplementary Figure 1: Acquisition of data for analysis from TCGA and Penn data sets. (**a**) Summary overview of the methods of acquisition and analysis for the TCGA germline *BRCA* and non*BRCA* datasets. (**b**) Summary overview of the methods of acquisition and analysis for the Penn *BRCA* dataset. BAM: Binary Alignment/Map format; GDC: Genome Data Common; QC: quality control; TCGA; The Cancer Genome Atlas; IGV: Integrated Genome Viewer; WES: whole exome sequencing.

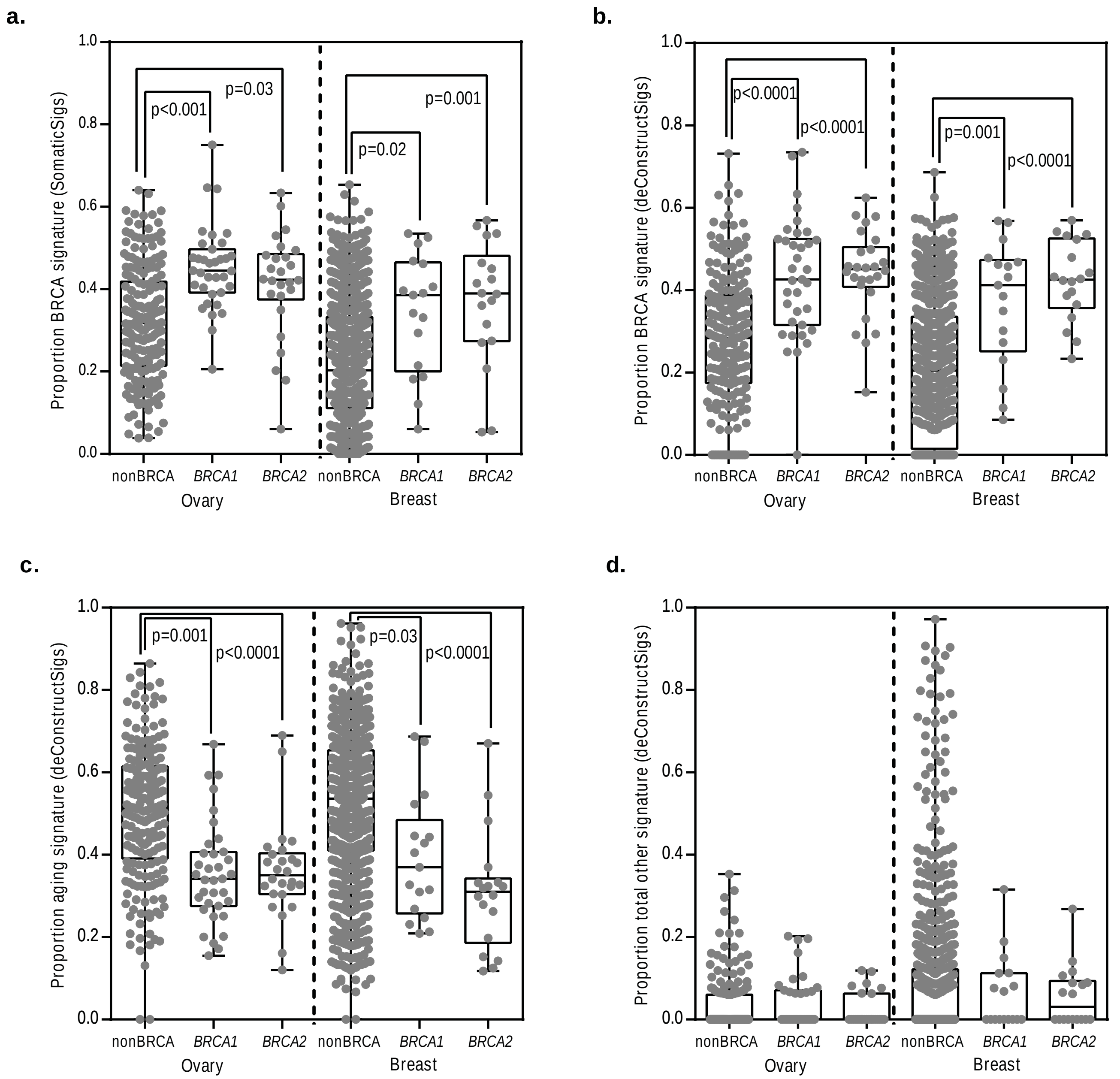

Supplementary Figure 2: Proportion of mutational signatures by mutation and tumor type in TCGA data. (a) Proportion of mutations due to *BRCA* mutational signature for *BRCA1* and *BRCA2* germline mutation-associated breast and ovarian tumors in the TCGA as compared to non*BRCA* tumors as analyzed by nonnegative matrix factorization (NMF) with the SomaticSigs program with r=4. **(b)** Proportion of mutations due to *BRCA* mutational signature for *BRCA1* and *BRCA2* germline mutation-associated breast and ovarian tumors in the TCGA as compared to nonBRCA tumors as analyzed by deconstructSigs. **(c)** Proportion of mutations due to aging mutational signature for *BRCA1* and *BRCA2* germline mutation-associated breast and ovarian tumors in the TCGA as compared to non*BRCA* tumors as analyzed by deconstructSigs. **(d)** Total proportion of mutations due to any other mutational signature for *BRCA1* and *BRCA2* germline mutation-associated breast and ovarian tumors in the TCGA as compared to non*BRCA* tumors as analyzed by deconstructSigs. For all boxplots, the center line represents the median, box limits are at the 25th and 75th percentile, and whisker limits are at the min and max of the measured value for the represented group. Three group continuous variable comparisons were performed using an ordinary one-way ANOVA with Tukey's multiple comparisons test with a single pooled variance. p-values are marked on the graphs for statistically significant comparisions, and all other comparsons are non-significant.

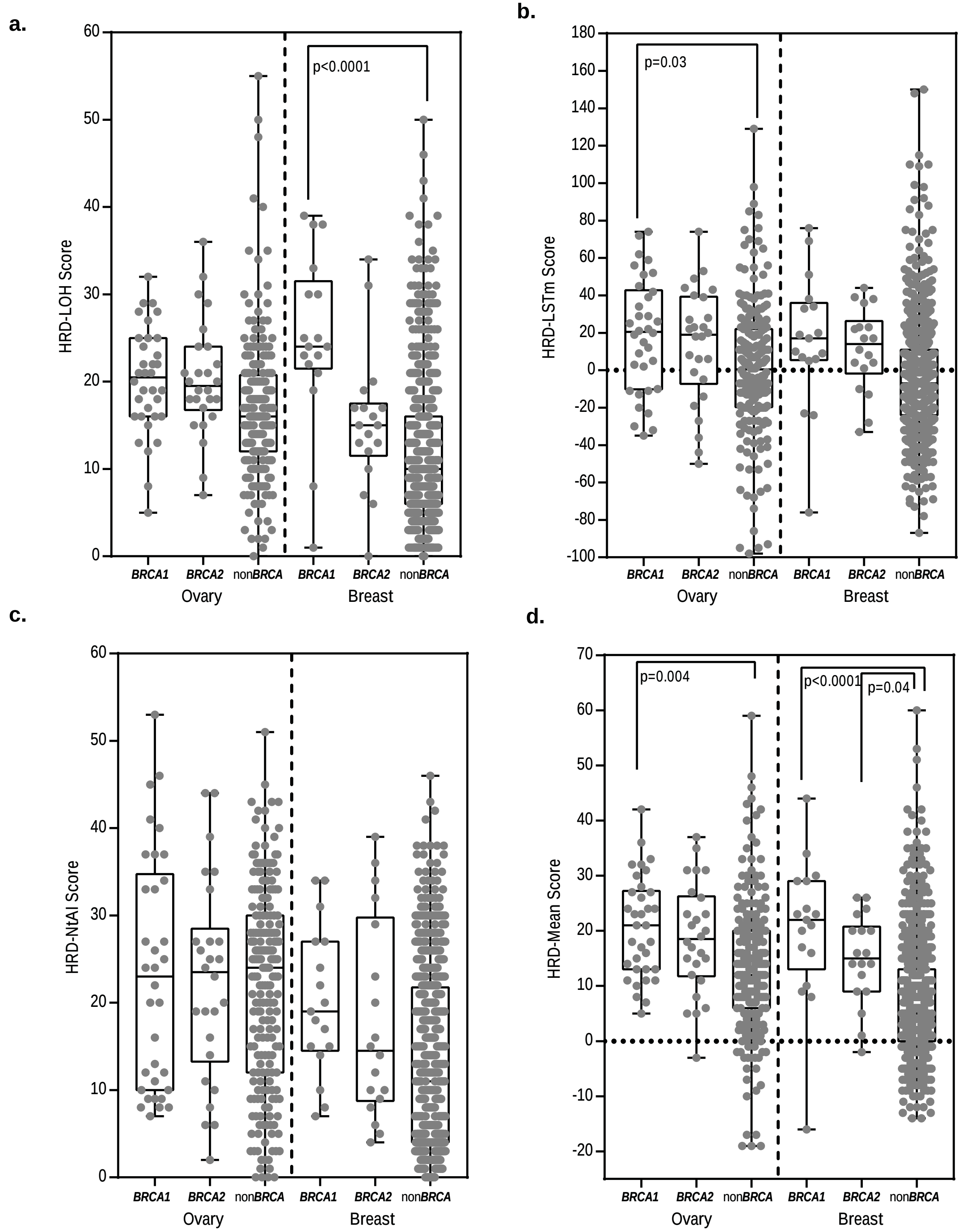

Supplementary Figure 3: HRD scores by mutation and tumor type using analyses of TCGA data. **(a)** HRD-LOH scores for germline *BRCA1* and *BRCA2* breast and ovarian tumors in the TCGA as compared to nonBRCA tumors. **(b)** HRD-LST scores for germline *BRCA1* and *BRCA2* breast and ovarian tumors in the TCGA as compared to nonBRCA tumors. **(c)** HRD-NtAI scores for germline *BRCA1* and *BRCA2* breast and ovarian tumors in the TCGA as compared to nonBRCA tumors. **(d)** HRD-Mean scores for germline *BRCA1* and *BRCA2* breast and ovarian tumors in the TCGA as compared to nonBRCA tumors. For all boxplots, the center line represents the median, box limits are at the 25th and 75th percentile, and whisker limits are at the min and max of the measured value for the represented group. Three group continuous variable comparisons were performed using an ordinary one-way ANOVA with Tukey's multiple comparisons test with a single pooled variance. p-values are marked on the graphs for statistically significant comparisions, and all other comparsons are non-significant. All comparisons between *BRCA1* and *BRCA2* subgroups were nonsignificant. HRD-LOH, HRD-NtAI and HRD-mean scores were significantly higher in nonBRCA ovary versus nonBRCA breast ($p<0.0001$) for all comparisons.

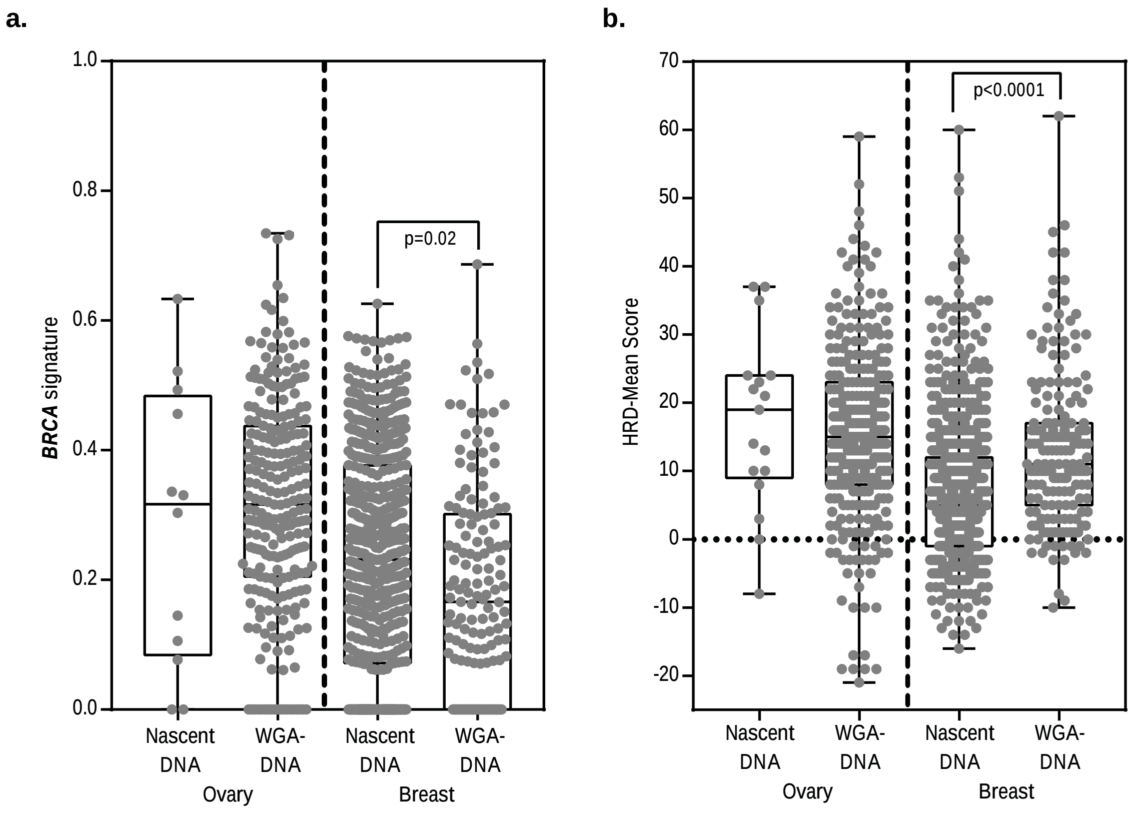

Supplementary Figure 4: *BRCA* mutational signature and HRD-Mean scores by analyte type in TCGA data. (a) Proportion of mutations due to *BRCA* mutational signature for TCGA breast and ovarian tumors as compared by analyte preparation method, nascent DNA versus whole genome amplified (WGA) DNA. **(b)** HRD-Mean scores for TCGA breast and ovarian tumors as compared by analyte preparation method, nascent DNA versus whole genome amplified (WGA) DNA. For all boxplots, the center line represents the median, box limits are at the 25th and 75th percentile, and whisker limits are at the min and max of the measured value for the represented group. Two group continuous variable comparisons were performed using a two-tailed Student's t-test. p-values are marked on the graphs for statistically significant comparisions, and all other comparsons are non-significant.

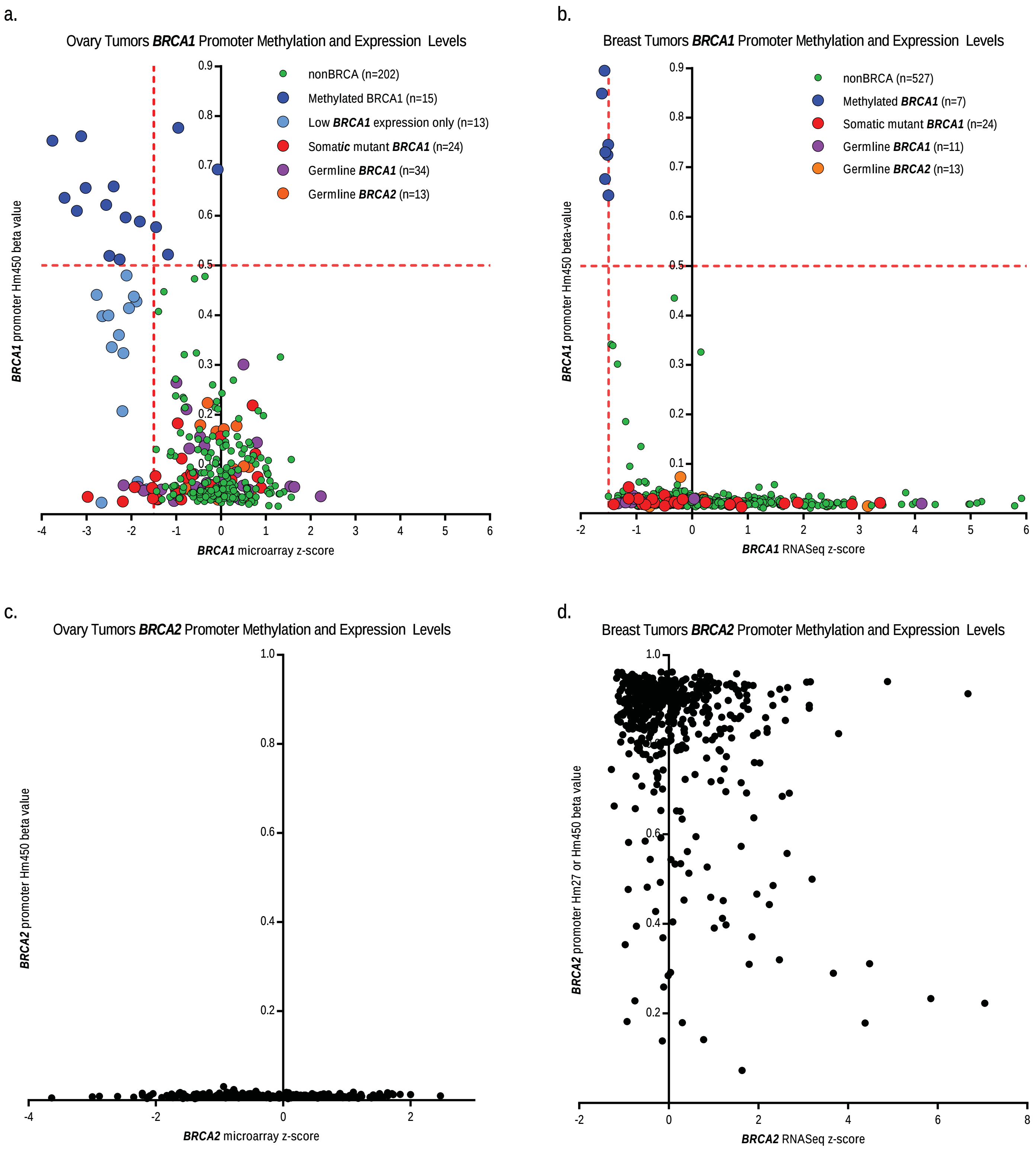

Supplementary Figure 5: Promoter methylation studies for *BRCA1* and *BRCA2* from TCGA breast and ovarian tumors. (**a**) *BRCA1* promoter Hm450 beta values plotted against *BRCA1* microarray z-scores for TCGA ovarian tumors where data was available for both values. (**b**) *BRCA1* promoter Hm450 beta values plotted against *BRCA1* RNASeq z-scores for TCGA breast tumors where data was available for both values. (**c**) *BRCA2* promoter Hm450 beta values plotted against *BRCA2* microarray z-scores for TCGA ovarian tumors where data was available for both values. (**d**) *BRCA2* promoter Hm450 beta values plotted against *BRCA2* RNASeq z-scores for TCGA breast tumors where data was available for both values.

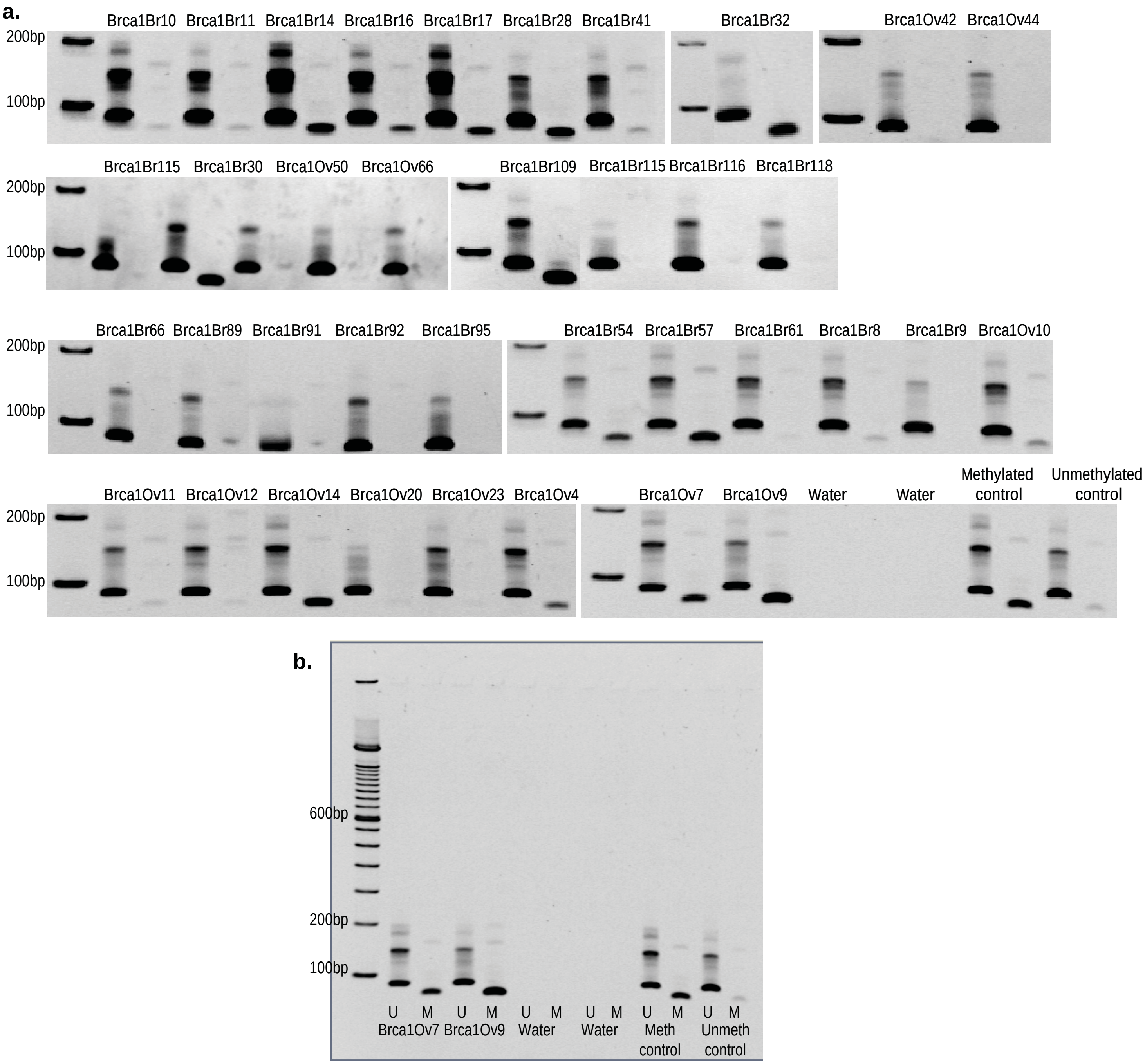

Supplementary Figure 6: Promoter methylation by methylation specific PCR for the *BRCA1* promoter in the Penn dataset. (**a**.) *BRCA1* methylation specific promoter studies for *BRCA1* germline mutation-associated tumors. Each tumor DNA is amplified with primer pair U (left set of bands) and primer pair M (right set of bands). Primer pair U amplifies unmethylated *BRCA1* promoter of an expected size of 86 base pairs (bp), and primer pair M amplifies methylated *BRCA1* promoter of an expected size of 75 base pairs, as shown for fully methylated and unmethylated control genomic DNA. The top ~150 base pair band amplified by the U primer pair is a nonspecific band. (b) Full representative gel image for Brca1Ov7, Brca1Ov9, water controls, methylated (meth) controls and unmethylated (unmeth) controls. Data shown representative of two independent PCR amplifications.

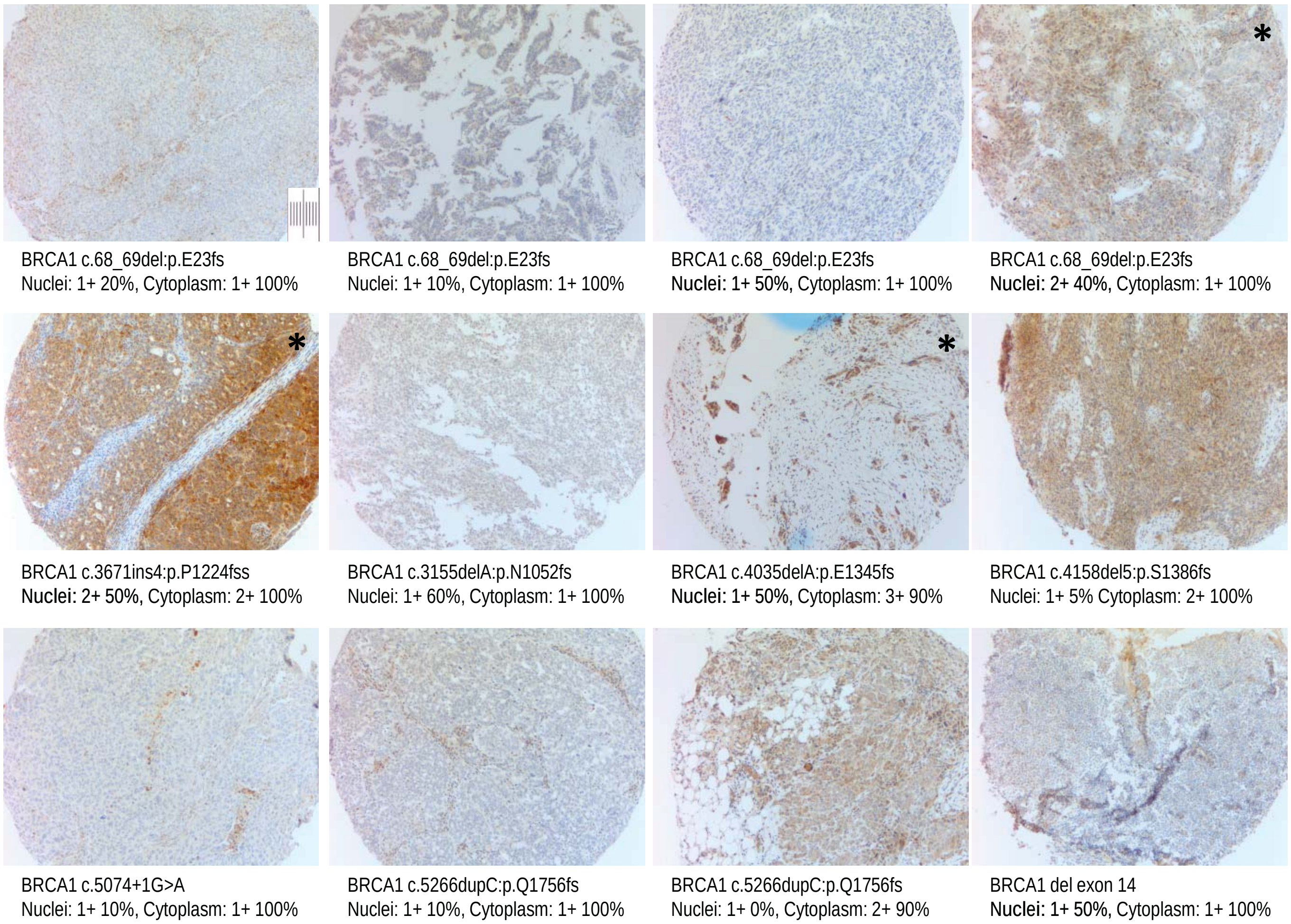

Supplementary Figure 7: Immunohistochemical staining for BRCA1 with antibody MS110 in *BRCA1* germline mutation-associated ovarian tumors. Staining was graded from 0 to 3+ in three cores in both tumor nuclei and normal tissue nuclei. One representative core with the maximum score for tumor nuclear staining is shown. Positive nuclear staining was defined as 100% of nuclei with at least 1+ staining in all three cores. Heterogeneous positive staining was defined as greater than 25% of nuclei with at least 1+ staining in at least one of three cores. ∗Tumors without locus-specific LOH. Scale bar represents 0.1mm at 0.01mm increments.

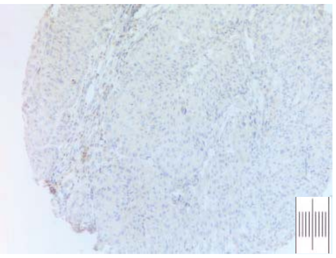

BRCA1 c.68_69del:p.E23fs
Nuclei: 0+ 100%, Cytoplasm: 0+, 100%

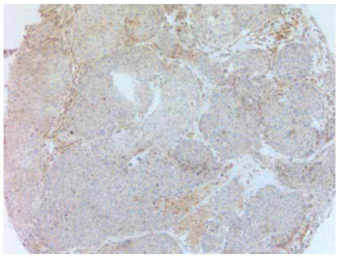

BRCA1 c.68_69del:p.E23fs
Nuclei: 0+ 100%, Cytoplasm 1+ 100%

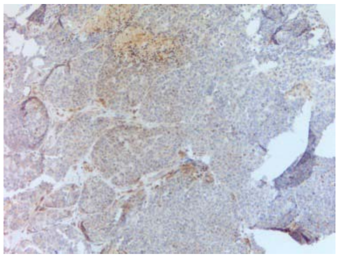

BRCA1 c.68_69del:p.E23fs
Nuclei: 0+ 100%, Cytoplasm 1+ 100%

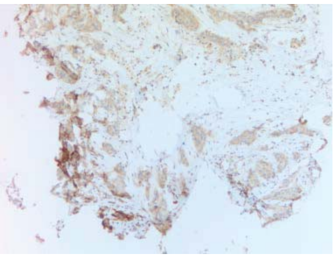

BRCA1 c.68_69del:p.E23fs
Nuclei: 0+ 100%, Cytoplasm 2+ 100%

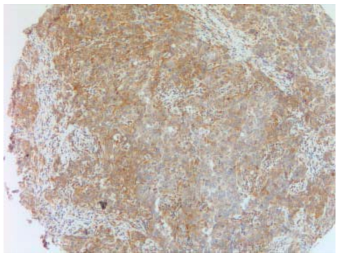

BRCA1 c.68_69del:p.E23fsLOHpos
Nuclei: 2+ 5%, Cytoplasm: 2+ 90%

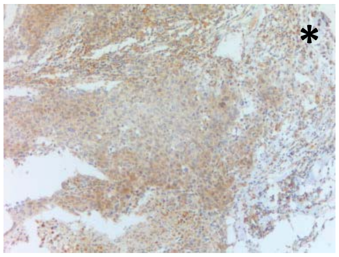

BRCA1 c.68_69del:p.E23fs
Nuclei:1+ 40%, Cytoplasm 1+ 100%

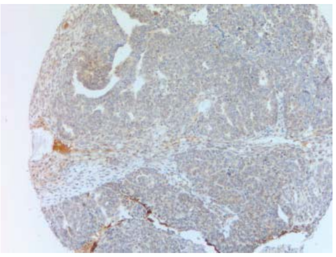

BRCA1 c.2197del5p.E733fs
Nuclei: 1+ 30%, Cytoplasm: 1+ 90%

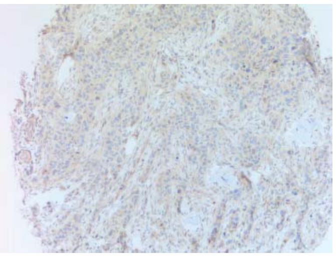

BRCA1 c.T2934G:p.Y978X
Nuclei: 0+ 100%, Cytoplasm 1+ 100%

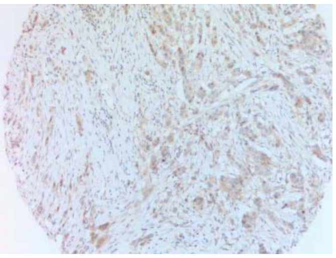

BRCA1 c.3756del4:p.S1253fs
Nuclei: 1+ 10%, Cytoplasm: 1+ 90%

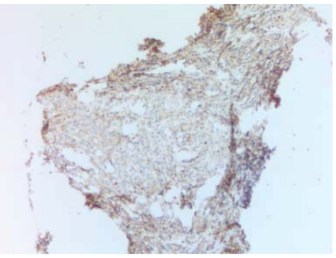

BRCA1 c.C4327T:p.R1443X
Nuclei: 0+ 100%, Cytoplasm 1+ 100%

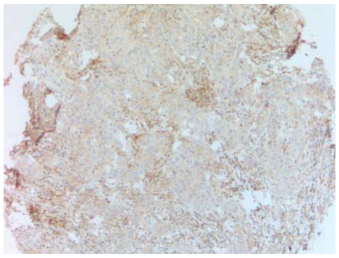

BRCA1 c.5074+1G>T
Nuclei: 0+ 100%, Cytoplasm: 1+ 100%

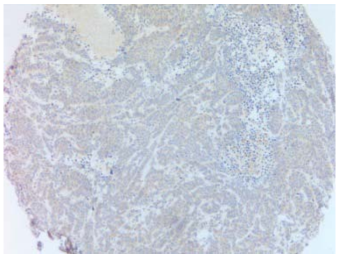

BRCA1 exon 14-20 del 26kb
Nuclei: 0+ 100%, Cytoplasm 1+ 100%

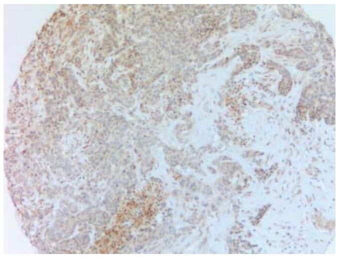

BRCA1 del exon 8
Nuclei: 2+ 10%, Cytoplasm: 2+ 5%

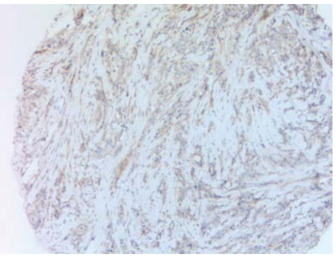

BRCA1 exon 13 ins 6kb
Nuclei: 0+ 100%, Cytoplasm 1+ 100%

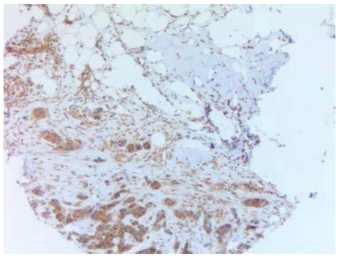

BRCA1 c.G5074C:p.D1692H
Nuclei: 1+ 50%, Cytoplasm: 2+ 100%

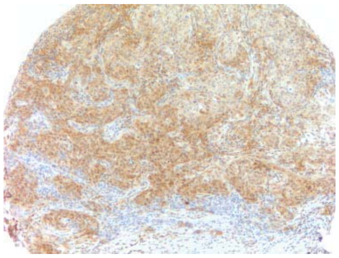

BRCA1 c.T5066G:p.M1689R
Nuclei: 1+ 30%, Cytoplasm: 2+ 50%

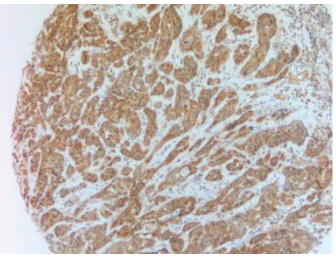

BRCA1 c.T181G:p.C61G
Nuclei: 1+ 10%, Cytoplasm: 2+ 90%

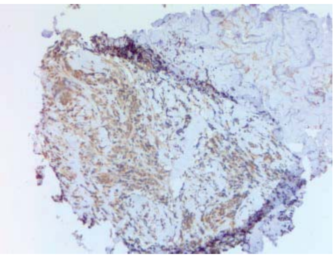

BRCA1 c.C5123A:p.A1708E
Nuclei: 0+ 100%, Cytoplasm: 2+ 90%

Supplementary Figure 8: Immunohistochemical staining for BRCA1 with antibody MS110 in *BRCA1* germline mutation-associated breast tumors. Staining was graded from 0 to 3+ in three cores in both tumor nuclei and normal tissue nuclei. One representative core with the maximum score for tumor nuclear staining is shown. Positive nuclear staining was defined as 100% of nuclei with at least 1+ staining in all three cores. Heterogeneous positive staining was defined as greater than 25% of nuclei with at least 1+ staining in at least one of three cores. ∗Tumors without locus-specific LOH. Scale bar represents 0.1mm at 0.01mm increments.

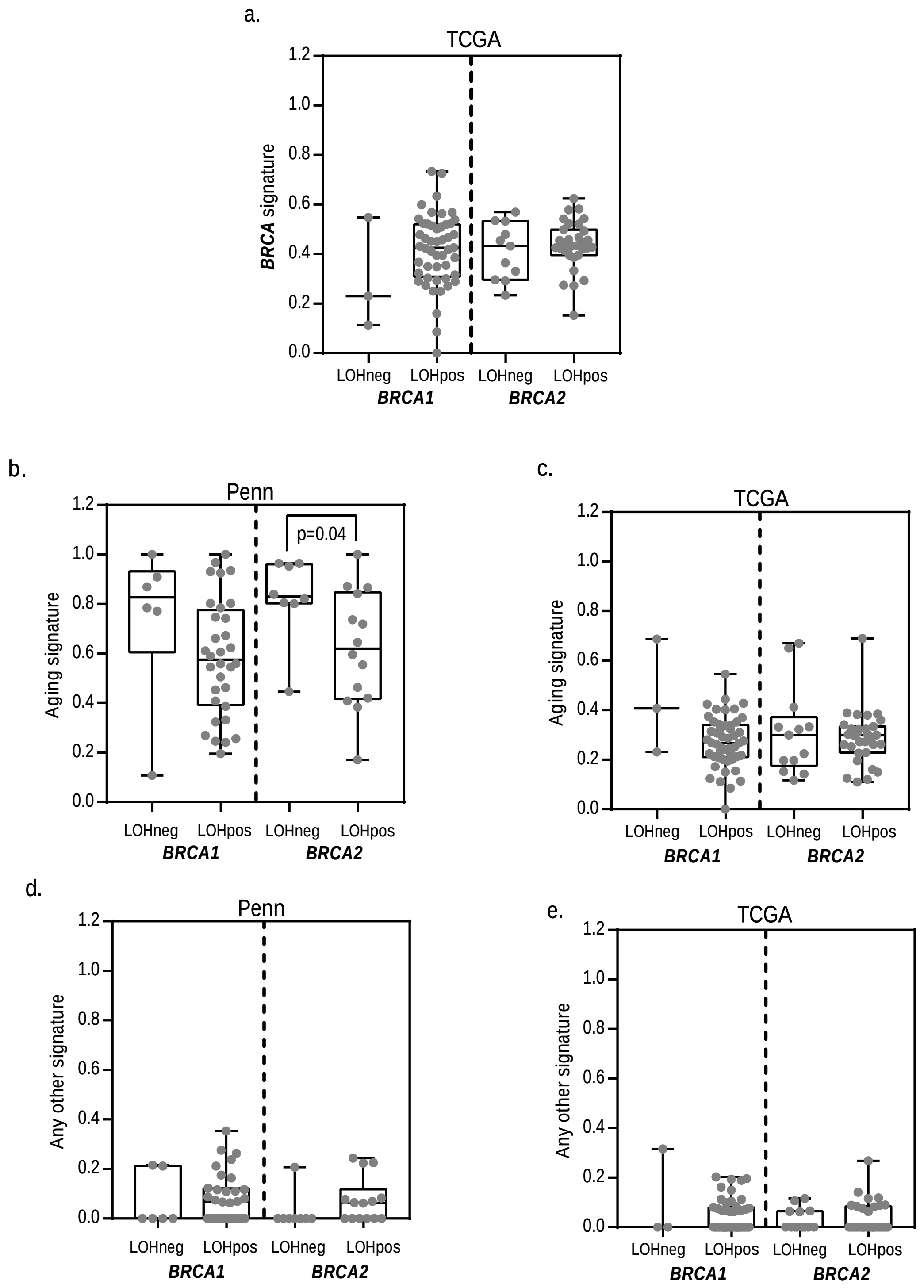

Supplementary Figure 9: Proportion of mutational signatures in Penn and TCGA germline *BRCA1* and *BRCA2* breast and ovarian tumors with and without locus- specific LOH. (a) Proportion of mutations due to *BRCA* mutational signature for germline *BRCA1* and *BRCA2* breast and ovarian tumors in the TCGA dataset. **(b)** Proportion of mutations due to aging mutational signature for germline *BRCA1* and *BRCA2* breast and ovarian tumors in the Penn dataset. **(c)** Proportion of mutations due to aging mutational signature for germline *BRCA1* and *BRCA2* breast and ovarian tumors in the TCGA dataset. **(d)** Proportion of mutations due to any other mutational signature for germline *BRCA1* and *BRCA2* breast and ovarian tumors in the Penn dataset. **(e)** Proportion of mutations due to any other mutational signature for germline *BRCA1* and *BRCA2* breast and ovarian tumors in the TCGA dataset. For all boxplots, the center line represents the median, box limits are at the 25th and 75th percentile, and whisker limits are at the min and max of the measured value for the represented group. Two group continuous variable comparisons were performed using a two-tailed Student's t-test. p-values are marked on the graphs for statistically significant comparisions, and all other comparsons are non-significant.

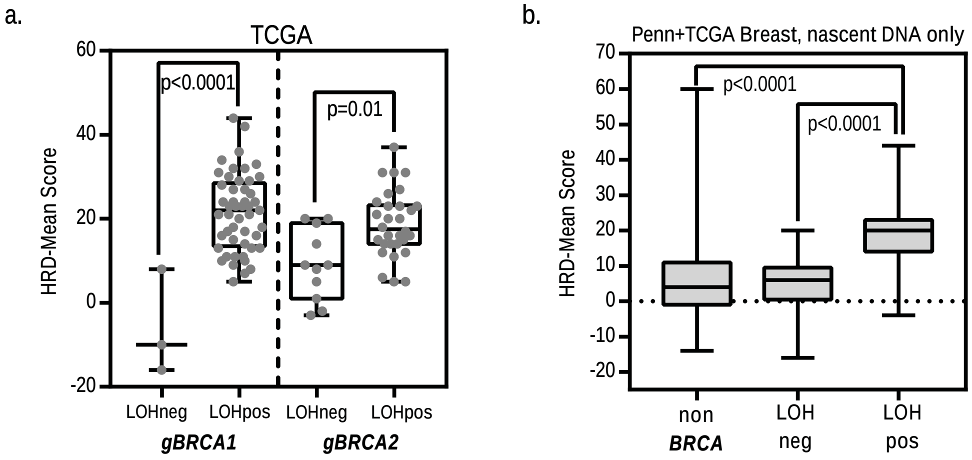

Supplementary Figure 10: Association of HRD-Mean scores with locus- specific LOH. (**a**) HRD-Mean scores in *BRCA1* and *BRCA2* tumors with (LOHpos) and without (LOHneg) locus-specific LOH in the TCGA data set. (**b**) HRD-Mean score comparison for combined Penn and TCGA dataset breast tumors derived from nascent DNA and excluding those derived from whole genome amplification (WGA). For all boxplots, the center line represents the median, box limits are at the 25th and 75th percentile, and whisker limits are at the min and max of the measured value for the represented group. Two group continuous variable comparisons were performed using a two-tailed Student's t-test. Three group continuous variable comparisons were performed using an ordinary one-way ANOVA with Tukey’s multiple comparisons test with a single pooled variance. p-values are marked on the graphs for statistically significant comparisions, and all other comparsons are non-significant.

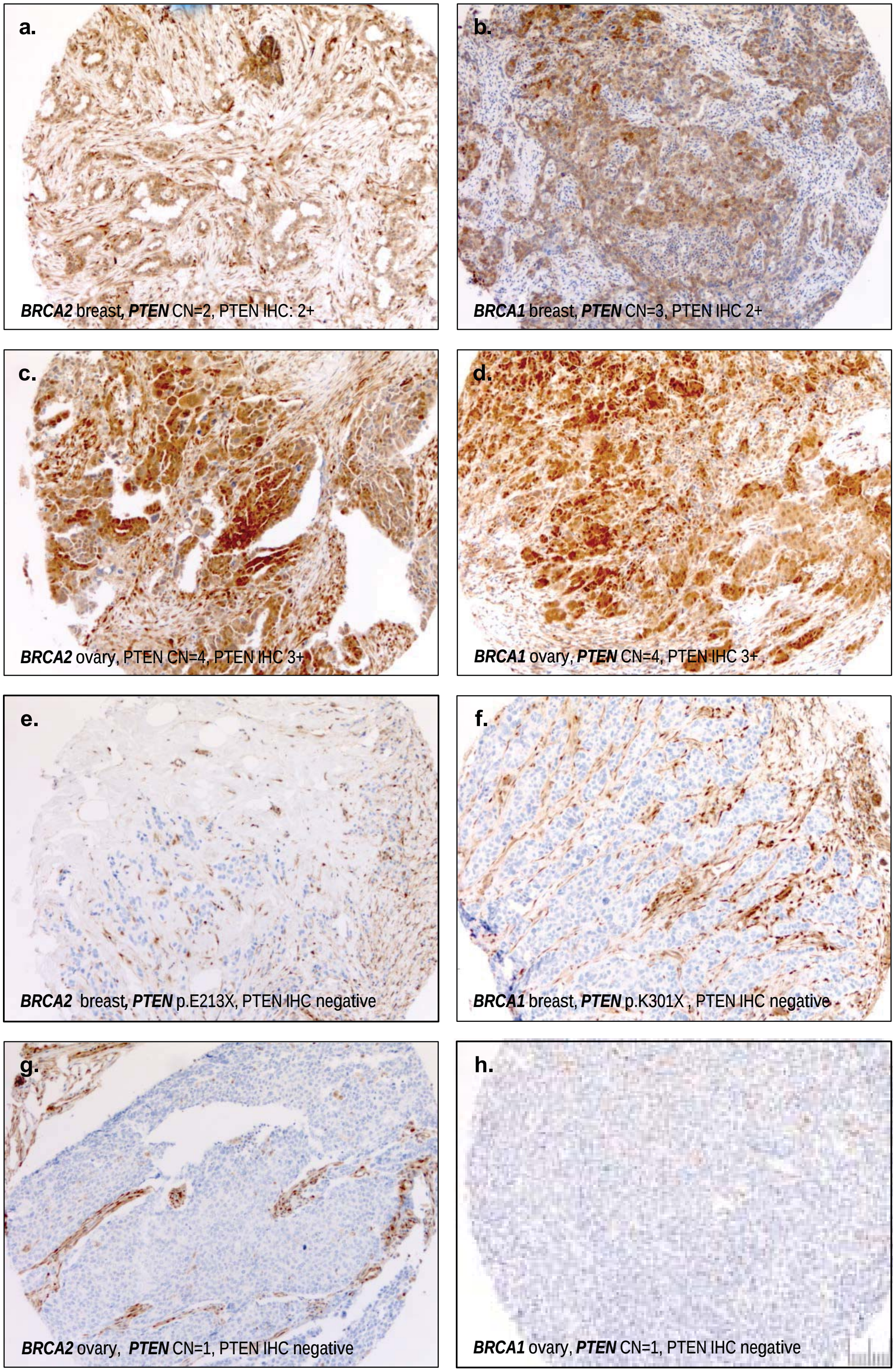

Supplementary Figure 11: Correlation between PTEN genomic analysis and immunohistochemistry. Representative images of immunohistochemistry (IHC) for PTEN in breast and ovarian tumors from patients with germline *BRCA1* or *BRCA2* mutations. (**a**). *BRCA2* breast tumor with copy number (CN) = 2 and wildtype *PTEN*, IHC score 2+. (**b**) *BRCA1* breast tumor with CN=3 and wildtype *PTEN*, IHC score 2+. (**c**) *BRCA2* ovary tumor with CN=4 and wildtype *PTEN*, IHC score 3+. (**d**) *BRCA1* ovary with CN=4 and wildtype *PTEN*, IHC score 3+. (**e**) *BRCA2* breast with a loss of function mutant *PTEN*, IHC score 0. (**f**) *BRCA1* breast with a loss of function mutant *PTEN*, IHC score 0**.** (**g**) *BRCA2* ovary with *PTEN* copy number loss (CN=1), IHC score 0**.** (**h**) *BRCA1* ovary with *PTEN* copy number loss (CN=1), IHC score 0. Scale bar represents 0.1mm at 0.01mm increments.

a.

Cox Proportional Hazards Model – Ovarian LOHneg vs LOHpos vs nonBRCA

Covariate	Status	Hazard Ratio	95% CI	P-value
*BRCA	LOHpos	0.37	0.17-0.79	0.0116
BRCA	nonBRCA	0.79	0.38-1.68	0.55
Site	TCGA	1.40	0.69-2.84	0.34
Stage	II	0.23	0.02-2.14	0.20
Stage	III	0.45	0.06-3.59	0.45

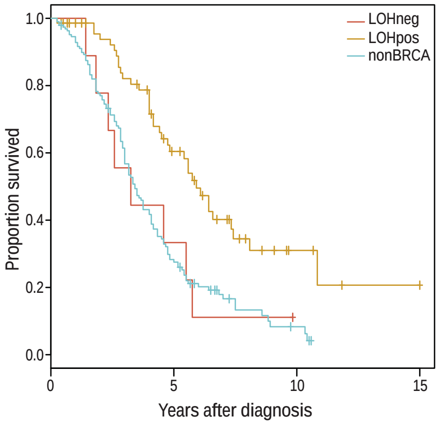

b.

Cox Proportional Hazards Model – Ovarian LOHpos vs LOHneg

Covariate	Status	Hazard Ratio	95% CI	P-value
BRCA	BRCA2	0.90	0.48-1.71	0.76
*LOH	LOHpos	0.33	0.14-0.74	0.00753
Site	TCGA	1.43	0.69-2.99	0.33
Stage	II	0.17	0.013-2.26	0.18
Stage	III	0.90	0.04-3.10	0.35

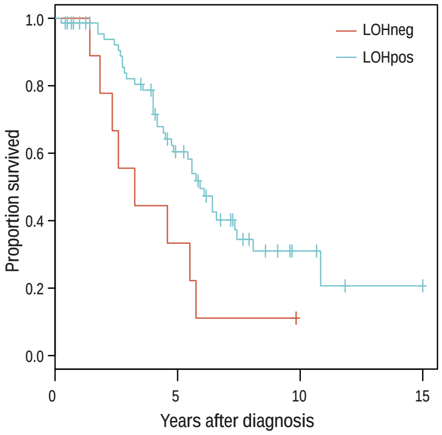

c.

Cox Proportional Hazards Model – Breast LOHneg vs LOHpos vs nonBRCA

Covariate	Status	Hazard Ratio	95% CI	P-value
*BRCA	nonBRCA	12.08	0.82-176.42	0.034
BRCA	LOHpos	3.76	0.46-30.80	0.22
Site	TCGA	0.45	0.05-3.76	0.45
Stage	II	1.72	0.84-3.47	0.13
*Stage	III	6.11	2.93-12.75	1.44e-06
HR Status	Her2+	1.29	0.75-2.22	0.36
HR Status	TNBC	1.94	1.12-3.63	0.01

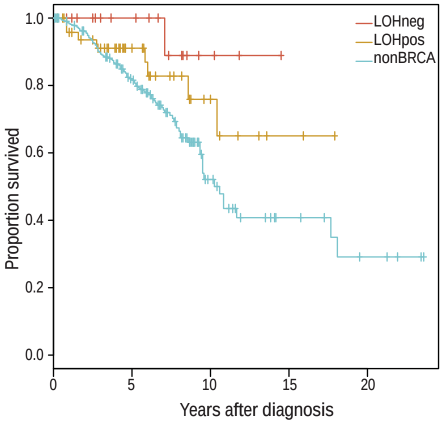

Supplementary Figure 12: Overall survival based on locus specific LOH status in patients with germline *BRCA1* and *BRCA2* mutations using Cox proportional hazard models. (a) Multivariate analysis using a Cox proportional hazards model comparing *BRCA1* and *BRCA2* germline mutation-associated ovarian tumors without locus specific LOH (LOHneg) to *BRCA1* and *BRCA2* germline mutation-associated ovarian tumors with locus-specific LOH (LOHpos) and nonBRCA patients in the TCGA and Penn dataset. **(b)** Multivariate analysis using a Cox proportional hazards model comparing *BRCA1* and *BRCA2* germline mutation-associated ovarian tumors without locus specific LOH (LOHneg) to *BRCA1* and *BRCA2* germline mutation-associated ovarian tumors with locus-specific LOH (LOHpos) in the TCGA and Penn dataset. **(c)** Multivariate analysis using a Cox proportional hazards model comparing *BRCA1* and *BRCA2* germline mutation-associated breast tumors without locus specific LOH (LOHneg) to *BRCA1* and *BRCA2* germline mutation-associated breast tumors with locus-specific LOH (LOHpos) and nonBRCA patients in the TCGA and Penn dataset.

Supplementary Table 1: Clinical characteristics of the Penn and TCGA dataset population

	Penn germline *BRCA* (n=60)		TCGA germline *BRCA* (n=100)		TCGA non*BRCA* (n=979)	
**Gender**						
Female	59	*98%*	99	*99%*	960	*98%*
**Germline Mutation**						
BRCA1	38	*63%*	55	*55%*	0	*0%*
BRCA2	22	*37%*	45	*45%*	0	*0%*
**Site**						
Breast	39	*65%*	37	*37%*	764	*78%*
Ovary	21	*35%*	63	*63%*	215	*22%*
**Tumor classification**						
Primary	51	*85%*	100	*100%*	979	*100%*
Second primary	9	*15%*	0	*0%*	0	*0%*
**Age of cancer diagnosis**						
Breast	44.7±9.7		48.8±12.6		59.0±13.0	
Ovary	54.3±7.2		52.7±9.9		61.6±11.1	
**Prior Chemotherapy**						
For another primary cancer	9	*15%*	0	*0%*	0	*0%*
**Breast Histology**						
Invasive ductal carcinoma	33	*85%*	29	*78%*	547	*72%*
Invasive lobular carcinoma	1	*3%*	5	*14%*	149	*20%*
Other or Unknown	5	*13%*	3	*8%*	68	*9%*
**Breast Hormone Receptors**						
TNBC	19	*49%*	11	*30%*	108	*14%*
ER+Her2-	15	*38%*	13	*35%*	416	*54%*
ER+Her2+	3	*8%*	4	*11%*	111	*15%*
ER-Her2+	1	*3%*	3	*8%*	32	*4%*
Unknown ER or HER2	1	*3%*	6	*16%*	97	*13%*
**Breast Stage**						
Stage IA/B/C	18	*46%*	2	*5%*	127	*17%*
Stage IIA/IIB	18	*46%*	24	*65%*	441	*58%*
Stage IIIA/B/C	3	*8%*	9	*24%*	170	*22%*
Stage IV	0	*0%*	0	*0%*	11	*1%*
Unstaged	0	*0%*	2	*5%*	15	*2%*
**Ovary Histology**						
Epithelial ovarian cancer	19	*90%*	63	*100%*	215	*100%*
Carcinosarcoma	2	*10%*	0	*0%*	0	*0%*
**Ovarian Cancer Stage**						
Stage I/II	5	*24%*	3	*5%*	12	*6%*
Stage III	11	*52%*	48	*76%*	165	*77%*
Stage IV	3	*14%*	10	*16%*	37	*17%*
Unstaged	1	*5%*	2	*3%*	1	*0%*
**DNA Preparation for Sequencing**						
Nascent DNA	60	*100%*	33	*33%*	581	*59%*
Whole Genome Amplified	0	*0%*	67	*67%*	398	*41%*

Supplementary Table 2: Details on individual tumors without locus specific LOH

Tumor	Gene	Mutation	Subtype	Age of dx	2nd hit[1]	BRCA1 IHC[2]	HRD-Mean[3]	Aging Sig	*BRCA* Sig	Other Mut Sigs	Mut Load[5]	TP53; PTEN Status[6]
Brca1Br11	*BRCA1*	p.E23fs	TNBC	39	No	+/+	9↓	0.87	0.12↓	none	1.08↑	Mut; +
Brca1Br9	*BRCA1*	p.E23fs	TNBC	55	No	nd	11↓	0.77	0.18↓	none	1.32↑	Mut; -
Brca1Br16	*BRCA1*	c.5074+1G>T	ER+Her2- IDC	40	No	0/+	2↓	0.78	0.00↓	2 (0.12)	0.06↓	WT; -
AR-A1AM	*BRCA1*	p.N1355fs	ER+Her2- ILC	52	No	n/a	-16↓	0.69	0.23↓	none	2.86↑	WT; +
A1-A0SH	*BRCA1*	p.C61G	TNBC	39	SM[7]	n/a	8↓	0.23	0.11↓	2 (0.32)	2.52↓	WT; +
Brca1Ov13	*BRCA1*	p.E1345fs	Ovarian HGPSC	53	No	0/+++	3↓	1.00	0.00↓	none	1.02↑	WT; +
Brca1Ov7	*BRCA1*	p.E23fs	Ovarian MMMT	57	PM	+/+	1↓	0.79	0.00↓	2 (0.13)	0.28↓	Mut; -
Brca1Ov9	*BRCA1*	p.P1224fs	Ovarian HGPSC	53	PM	++/++	9↓	0.91	0.00↓	none	0.92↓	Mut; +
13-0883	*BRCA1*	p.Q1756fs	Ovarian HGPSC	61	No	n/a	-10↓	0.41	0.55↑	none	2.20↓	WT; +
Brca2Br8	*BRCA2*	p.L2253fs	ER+Her2- IDC	37	No	n/a	-2↓	0.96	0.00↓	none	0.76↓	WT; +
Brca2Br21	*BRCA2*	c.8487+1G>A	ER+Her2- IDC	39	No	n/a	7↓	0.96	0.00↓	none	0.76↓	WT; -
Brca2Br11	*BRCA2*	p.Y1655X	ER+Her2- IDC	46	No	n/a	9↓	0.82	0.17↑	none	0.50↓	WT; +
Brca2Br7	*BRCA2*	p.Y91fs	ER+Her2- IDC	33	No	n/a	18↑	0.80	0.09↓	none	0.52↓	WT; +
Brca2Br3	*BRCA2*	p.Y352fs	ER+Her2- IDC	50	No	n/a	-1↓	0.45	0.00↓	13 (0.14)	0.56↓	WT; +
Brca2Br32	*BRCA2*	p.N2879fs	ER+Her2- ILC	52	No	n/a	5↓	0.95	0.00↓	none	0.26↓	WT; -
Brca2Br1	*BRCA2*	p.S1982fs	TNBC	70	No	n/a	2↓	0.81	0.00↓	none	0.30↓	WT; +
A8-A09A	*BRCA2*	p.E1953X	ER+Her2- IDC	40	No	n/a	20↑	0.20	0.54↑	none	5.56↑	WT; +
AO-A03V	*BRCA2*	p.S1982fs	ER+Her2- IDC	41	No	n/a	20↑	0.14	0.43↑	none	1.92↓	Mut; -
BH-A0AZ	*BRCA2*	p.S1955X	ER+Her2- IDC	47	No	n/a	14↓	0.32	0.36↓	2 (0.12)	1.62↓	WT; +
D8-A1JD	*BRCA2*	p.P2283fs	ER+Her2- IDC	41	No	n/a	5↓	0.12	0.57↑	none	4.08↑	WT; +
E9-A1NE	*BRCA2*	p.T1325fs	ER+Her2- IDC	28	No	n/a	9↓	0.54	0.30↓	none	1.38↓	WT; -
AC-A3BB	*BRCA2*	p.T219fs	ER+Her2- ILC	46	No	n/a	1↓	0.67	0.23↓	none	27.4↑	WT; +
EW-A1PD	*BRCA2*	p.T256fs	ER+Her2+ IDC	61	No	n/a	9↓	0.33	0.48↑	none	3.00↑	WT; +
EW-A1P7	*BRCA2*	p.D2161fs	TNBC	59	No	n/a	-2↓	0.15	0.53↑	none	2.02↓	WT; +
A2-A0T0	*BRCA2*	p.T2314fs	TNBC	59	SM[8]	n/a	26↑	0.33	0.44↑	none	4.54↑	WT; -
Brca2Ov13	*BRCA2*	p.Q1056fs	Ovarian HGPSC	53	No	n/a	0↓	0.84	0.16↑	none	0.02↓	WT; +
24-0975	*BRCA2*	c.631+2T>G	Ovarian HGPSC	58	No	n/a	8↓	0.40	0.45↑	none	2.50↓	Mut; +
24-2293	*BRCA2*	p.R2520X	Ovarian HGPSC	47	No	n/a	-3↓	0.65	0.29↓	none	2.32↓	Mut; +
13-0751	*BRCA2*	p.L1521fs	Ovarian HGPSC	44	No	n/a	19↑	0.41	0.33↓	10 (0.06)	1.52↓	Mut; +
13-1498	*BRCA2*	p.S1982fs	Ovarian HGPSC	73	*[9]	n/a	35↑	0.30	0.56↑	none	3.20↑	Mut; -

[1]SM: somatic mutation, PM:promoter methylation
[2]IHC score see Table X
[3]HRD-Mean for tumors with locus-specific LOH (Mean±SD): Penn *BRCA1*: 21±2; Penn *BRCA2*: 15±3; TCGA *BRCA1*: 21±9; TCGA *BRCA2*: 19±8
[4]*BRCA* signature for tumors wtih locus-specifc LOH (Mean±SD): Penn *BRCA1*:0.20±0.13; Penn *BRCA2*:0.16±0.12; TCGA *BRCA1*:0.42±0.15; TCGA *BRCA2:* 0.43±0.09
[5]Mutational burden for tumors wtih locus-specifc LOH (Mean+SD): Penn *BRCA1*: 1.00+0.37; Penn *BRCA2*:1.45+1.08; TCGA *BRCA1*:2.80+0.97; TCGA *BRCA2*: 2.69+1.10
[6]*TP53* status is either mutant (mut) or wildtype (WT); PTEN status is either lost or retained
[7]A1-A0SH also has somatic *BRCA1* p.Q934X at 21% AF
[8]A2-A0T0 tumor also has somatic *BRCA2* p.L2926X at 35% AF
[9]13-1498 also has a germline *RAD51D* mutation with locus-specific LOH in the tumor

Supplementary Table 3: Somatic mutations identified in *BRCA1* and *BRCA2* germline mutation-associated tumors

Tumor Class[1]	≥1 mutation		DNA repair, Chromatin			Structural			Cell cycle			Oncogenic signaling		
	n	%	n	%	Genes	n	%	Genes	n	%	Genes	n	%	Genes
**BRCA1 Breast**														
LOHneg (n=5)	3	60%	0	0%	-	1	20%	*MYO5A*	0	0%	-	2	40%	*BMPR1A, PTEN*
LOHpos (n=35)[1]	12	34%	6	17%	*KDM6A(2), BLM, DNMT3A, ERCC6, NCOR2*	3	9%	*CDH15, FAT1, NUP98, PPFIBP1*	0	0%	-	7	20%	*NF1 (2), BCL2L2, CREBBP, FGFR2, NOTCH2, PIK3C2G*
**BRCA1 Ovary**														
LOHneg (n=4)	0	0%	0	0%	-	0	0%	-	0	0%	-	0	0%	-
LOHpos (n=46)[1]	25	54%	11	24%	*ARID1A(2), APEX1, DDB1, ERCC6, LIG4, NSD1, PARP4, POLR2A, POLM, TDG, UBR5*	3	7%	*COL7A1, FAT3*	3	7%	*RB1 (2), MITF*	14	30%	*NF1 (3), ABL1, BTK, CHUK, DAXX, JAK3, KRAS, MAP3K13, MAP3K15, MTOR, PIK3CA, PRKCG, PTEN*
**BRCA2 Breast**														
LOHneg (n=16)[1]	3	19%	1	6%	*POLR2A, SMARCA4*	2	13%	*EXT1, NUP98, NVASC*	0	0%	-	2	13%	*CDK12, PIK3CA*
LOHpos (n=18)	12	67%	6	33%	*ARID1A (2), KMT2C, KMT2D, MRE11A, NCOR1, TDG*	2	11%	*CDH1, FAT3, ITGAV*	4	22%	*RB1 (3), CDC27*	7	39%	*PIK3CA(2), AKT1, MAP2K4, MAP3K13, NOTCH2, NTRK2, SF1*
**BRCA2 Ovary**														
LOHneg (n=5)	2	40%	3	60%	*RPA2, FANCL, TOP3A*	0	0%	-	0	0%	-	1	20%	*NF1*
LOHpos (n=27)	14	52%	9	33%	*ATRX (2), ERCC6,FANCD2, FANCE, KMT2A, KMT2C, MSH2, SETDB1*	2	7%	*CDH15, NFASC*	4	15%	*RB1 (2), BCL6, CDC27*	4	15%	*NF1 (2), AXIN2, EPHB1, ZFHX3*

[1]One *BRCA2* breast tumor without locus-specific LOH, one *BRCA1* breast tumor with locus-specific LOH, and two *BRCA1* ovarian tumors with locus-specific LOH were excluded due to having >30% of their mutational signature due to sequencing artifact (all in TCGA dataset).

Supplementary Table 4: Confirmation of *PTEN* copy number calls by immunohistochemistry

**Copy number status at *PTEN* genomic locus**	**n**	**PTEN IHC Positive**	**PTEN IHC Negative**	**Concordance**
Heterozygous diploid or copy number gain	25	24	1	96%
*C*opy number loss or mutation	10	0	10	100%
Copy neutral LOH	9	4	5	n/a

Supplementary Table 5: Clinical correlates with *BRCA* locus specific LOH

	***BRCA1* breast**				***BRCA2* breast**			
Locus specific LOH	Present		Absent		Present		Absent	
n	36		5		18		16	
Avg Age diagnosis (± SD)	46.2±11.2		43.4±9.9		49.2±15.2		47.8±9.8	
Node positive (n, %)	6	17%	1	20%	12	67%	11	69%
ER+ (n, %)	9	26%	2	40%	12	67%	13	81%
HER2+ (n, %)	6	17%	0	0%	3	17%	3	19%
TNBC (n, %)	21	60%	3	60%	3	17%	3	19%
Deceased	6	17%	0	0%	1	6%	1	6%

	***BRCA1* ovary**				***BRCA2* ovary**			
Locus specific LOH	Present		Absent		Present		Absent	
n	48		4		27		5	
Avg Age diagnosis (± SD)	51.0±8.0		57.0±4.6		56.0±10.6		55.8±11.4	
Grade 1 or 2 (n, %)	8	17%	0	0%	1	4%	0	0%
Deceased	25	52%	3	75%	10	37%	5	100%
